# Resolving Differential Diagnostic Problems in von Willebrand Disease, in Fibrinogen Disorders, in Prekallikrein Deficiency and in Hereditary Hemorrhagic Telangiectasia by Next-Generation Sequencing

**DOI:** 10.3390/life11030202

**Published:** 2021-03-05

**Authors:** Réka Gindele, Adrienne Kerényi, Judit Kállai, György Pfliegler, Ágota Schlammadinger, István Szegedi, Tamás Major, Zsuzsanna Szabó, Zsuzsa Bagoly, Csongor Kiss, János Kappelmayer, Zsuzsanna Bereczky

**Affiliations:** 1 Division of Clinical Laboratory Science and Specialist Clinical Hemostasis Laboratory, Department of Laboratory Medicine, Faculty of Medicine, University of Debrecen, 4032 Debrecen, Hungary; gindele.reka@med.unideb.hu (R.G.); kallai.judit@med.unideb.hu (J.K.); szabo.zsuzska@med.unideb.hu (Z.S.); bagoly@med.unideb.hu (Z.B.); 2 Department of Laboratory Medicine, Faculty of Medicine, University of Debrecen, 4032 Debrecen, Hungary; kerenyi@med.unideb.hu (A.K.); kappelmayer@med.unideb.hu (J.K.); 3 Department of Laboratory Medicine, Faculty of Pharmacy, University of Debrecen, 4032 Debrecen, Hungary; 4 Center of Expertise for Rare Diseases, Clinical Center, University of Debrecen, 4032 Debrecen, Hungary; pfliegler@med.unideb.hu; 5 Department of Internal Medicine, Faculty of Medicine, University of Debrecen, 4032 Debrecen, Hungary; schlammadinger.agota@med.unideb.hu; 6 Division of Pediatric Hematology and Oncology, Department of Pediatrics, Faculty of Medicine, University of Debrecen, 4032 Debrecen, Hungary; iszegedi@med.unideb.hu (I.S.); kisscs@med.unideb.hu (C.K.); 7 Otorhinolaryngology and Head-Neck Surgery Division, Kenézy Gyula Teaching Hospital, University of Debrecen, 4032 Debrecen, Hungary; major.tamas@kenezy.unideb.hu

**Keywords:** next generation sequencing, rare bleeding disorders, von Willebrand disease, hemophilia A, hereditary hemorrhagic telangiectasia, differential diagnosis, prekallikrein deficiency, APTT prolongation

## Abstract

Diagnosis of rare bleeding disorders is challenging and there are several differential diagnostics issues. Next-generation sequencing (NGS) is a useful tool to overcome these problems. The aim of this study was to demonstrate the usefulness of molecular genetic investigations by summarizing the diagnostic work on cases with certain bleeding disorders. Here we report only those, in whom NGS was indicated due to uncertainty of diagnosis or if genetic confirmation of initial diagnosis was required. Based on clinical and/or laboratory suspicion of von Willebrand disease (vWD, *n* = 63), hypo-or dysfibrinogenemia (*n* = 27), hereditary hemorrhagic telangiectasia (HHT, *n* = 10) and unexplained activated partial thromboplastin time (APTT) prolongation (*n* = 1), NGS using Illumina platform was performed. Gene panel covered 14 genes (*ACVRL1*, *ENG*, *MADH4*, *GDF2*, *RASA1*, *F5*, *F8*, *FGA*, *FGB*, *FGG*, *KLKB1*, *ADAMTS13*, *GP1BA* and *VWF*) selected on the basis of laboratory results. We identified forty-seven mutations, *n* = 29 (6 novel) in vWD, *n* = 4 mutations leading to hemophilia A, *n* = 10 (2 novel) in fibrinogen disorders, *n* = 2 novel mutations in HHT phenotype and two mutations (1 novel) leading to prekallikrein deficiency. By reporting well-characterized cases using standardized, advanced laboratory methods we add new pieces of data to the continuously developing “bleeding disorders databases”, which are excellent supports for clinical patient management.

## 1. Introduction

Correct and detailed laboratory diagnosis of bleeding disorders is of key importance in order to offer the adequate therapeutic strategy to patients. According to the definition by the International Society of Thrombosis and Haemostasis (ISTH), all coagulopathies and platelet functional disorders with a prevalence lower than 1 in 500,000 people and the hereditary hemorrhagic telangiectasia (HHT) are considered as rare bleeding disorders, while hemophilia A (prevalence 1:5000 males), hemophilia B (prevalence 1:30,000 males) and von Willebrand disease (vWD) (prevalence 1:1000) are not included in the “Rare bleeding disorders” databases or registries, Rare Bleeding Disorders Network, [1]. However, certain subtypes of vWD (see below) have a sufficiently low frequency to be considered as rare [2,3]. Due to the low prevalence of congenital bleeding disorders the availability of experienced diagnostic laboratories is scarce.

Diagnosis of rare bleeding disorders is challenging and there are several differential diagnostic issues. In coagulation disorders laboratory screening tests are prothrombin time (PT), activated partial thromboplastin time (APTT) and thrombin time (TT) [4]. These clotting time assays are easy to perform and are widely available at a relatively low cost. Their prolongation, especially the pattern of prolonged clotting times (i.e., isolated clotting time prolongation vs. simultaneously prolonged values) represents the starting point for the diagnostic process of coagulopathies (except for factor XIII deficiency, where screening tests are normal). Differentiation among coagulation factor defects associated with isolated APTT prolongation is usually unequivocal since the determination of the intrinsic coagulation factor activities easily identify the one which is “missing”. To clarify the background of APTT prolongation, however, is not always such a simple issue. Apart from acquired causes, which are beyond the scope of this work, APTT prolongation may be caused by deficiencies of the so-called contact factors, as prekallikrein (PK), high molecular weight kininogen (HMWK) or factor XII [5]. They are now considered as clinically silent but may be associated with extreme APTT prolongation. Disorders of the PK-HMWK-bradykinin pathway may result in inflammatory signs and cardiovascular complications. By promoting tissue plasminogen activator release and activating pro-urokinase into urokinase, kallikrein plays a role in fibrinolysis [6,7]. Identifying PK or HMWK deficiency, therefore, may be important also from the point of view of further research to clarify their impact in different clinical settings [8]. Without clear confirmation of the nature of APTT prolongation the patient might be subjected to unnecessary disease alerts, and delay in surgical procedures and difficulties in anticoagulant therapy monitoring [9]. PK deficiency is inherited as an autosomal recessive (AR) trait and caused by mutations in the *KLKB1* gene.

vWD is in the major focus of discussion in clinical hemostasis laboratories. There are several differential diagnostic problems with a serious impact in patient management. Von Willebrand factor (vWF) is a huge multimeric glycoprotein and has multiple functions in hemostasis, which are related to different molecular regions and most pronounced under high blood shear rate conditions [10]. vWF supports platelet adhesion to the sub-endothelial collagen structures via platelet receptor glycoprotein Ib-alpha (GPIbα) and also promotes platelet aggregation via GPIIb-IIIa receptors. These bindings are related mainly to A1 and C4 domains of vWF, respectively. The presence of high molecular weight multimers (HMWM) and intact multimeric structure is of key importance for supporting platelet adhesion and collagen binding. The protein named as ADAMTS13 (A Disintegrin and Metalloproteinase with ThromboSpondin type 1 repeats, member 13) regulates multimeric structure of vWF by preventing the accumulation of ultra-large multimers [11]. vWF interacts with coagulation factor VIII (FVIII) in the circulation via the D’–D3 region of vWF, thus prolonging the half-life of FVIII and delivers it to the site of vascular injury [12]. According to its multiple functions vWD has different subtypes with different laboratory phenotype and with different therapeutic needs [13]. The most common symptoms in vWD in general are mucocutaneous bleedings, including heavy menstruation in females and re-bleeding after interventions [14,15]. The *VWF* gene is localized to chromosome 12p13.3 and contains 52 exons spanning approximately 178 kb [16]. Most types of vWD show autosomal dominant (AD) inheritance with a consequence of bleeding even in heterozygous subjects. Type 1 (AD) is a quantitative vWD associated with mild to severe bleeding, while type 3 vWD (AR), the most severe form is associated with a virtually absent vWF [3]. Type 2 vWD represents different functional defects. Type 2A (AD) is associated with the lack of large multimers and decreased affinity of vWF to bind to platelet GPIbα receptors. Type 2A is further subdivided according to the underlying molecular defect (mainly in A2, D2, D3 and CK domains) and the abnormality in multimeric structure to IIA, IIC, IID and IIE [17,18]. Type 2B (AD) associates with increased affinity of vWF to platelet receptor GPIbα with loss of large multimers; the molecular defect is in the A1 domain. If the enhanced platelet–vWF binding is due to mutations in platelet GPIbα the presence of platelet-type vWD is considered [19]. Type 2M (AD) is characterized by decreased platelet-vWF binding with normal multimeric structure having molecular defect mainly in the A1 or A3 domains of vWF. Finally, type 2N (AR) affects vWF-FVIII binding resulting in a decreased FVIII level and symptoms corresponding to hemophilia A. The mutations may be detected in the D’ or D3 domain in homozygous form, or patients may be compound heterozygotes with one mutation in the D’ or D3 domain and with another one elsewhere in *VWF* leading to null mutation [20].

Laboratory diagnosis of vWD is complex and only expert laboratories may offer the full spectrum of assays [21]. After evaluation of the bleeding history, laboratory work-up of vWD starts with screening assays of coagulation (APTT) and platelet numbers and function (PFA-100/200 closure times). As screening assays are neither sensitive nor specific enough for vWD the laboratory panel also includes the determination of vWF antigen level (vWF:Ag) and a vWF platelet-dependent activity assay. A functional assay may reflect the classical functional test in the presence of ristocetin and normal platelet preparation (ristocetin cofactor activity, vWF:RCo), or it may be performed in the presence of a monoclonal antibody directed against the GPIbα epitope of vWF (vWF:Ab), or platelets may be replaced by recombinant GPIbα and the test is performed in the presence of ristocetin (vWF:GPIbR), or a gain-of function mutant GPIbα is used, which—due to spontaneous binding to vWF—can exclude ristocetin (vWF:GPIbM). Finally, FVIII activity (FVIII:C) is also determined as first-line assay. A functional defect is suspected if the ratio of an activity assay result (vWF:Ac) to vWF:Ag is below 0.6. To classify vWD into the above-mentioned types further assays are needed, namely, collagen-binding assay (vWF:CB), FVIII-binding assay (vWF:FVIIIB), vWF multimeric analysis, ristocetin induced platelet aggregation (RIPA), which may discriminate enhanced vWF-platelet binding from decreased or normal vWF-platelet interaction by using a low and a high concentration of ristocetin in the test. Genotyping in vWD is not necessary in unequivocal cases; however, strengthening the biochemical diagnosis may be important also in such cases for genetic counseling, or in type 3 patients for prenatal diagnosis [21]. Apart from these situations genetic investigations in vWD may help to resolve several dilemmas. First of all, differentiation between hereditary and acquired vWD is crucial from the point of view of therapy. Additionally, due to the biological variability of vWF levels, it is sometimes hard to distinguish between real vWD and the so-called “low-vWF” category with inconsistent linkage to *VWF* [22]. As type 2 is a heterogeneous group, there are cases, in which biochemical assays cannot ensure the diagnosis.

Laboratory diagnosis of fibrinogen abnormalities is another interesting issue. Congenital fibrinogen disorders are rare and they can be either quantitative (hypo-or afibrinogenemia) or qualitative (dysfibrinogenemia, or hypo-dysfibrinogenemia) [23]. Severe quantitative defects are often associated with early bleeding episodes, while less severe ones may remain asymptomatic. Dysfibrinogenemia is more heterogeneous and may be associated with either bleeding or thrombosis, or may be asymptomatic. In the laboratory, determination of TT, which is routinely not performed in many laboratories, is a useful screening test. In the majority of cases TT is prolonged in hypo-, a-, dys-, and hypo-dysfibrinogenemia. According to the disease severity and the sensitivity of the reagents, PT and APTT can also be prolonged, moreover reptilase time (RT) is a good choice as it is highly prolonged in most of the cases [24,25]. In quantitative fibrinogen disorders functional fibrinogen (i.e., the Clauss method) and fibrinogen antigen are proportionally decreased, while in qualitative fibrinogen abnormalities the ratio of functional fibrinogen to fibrinogen antigen is low. The nature of the molecular defect may also have an impact on the results of the coagulation assays, as some mutations may not lead to clotting time prolongation in mechanical clot detection assays or in both methods (i.e., mechanical and optical clot detection). Fibrinogen disorders have a heterogeneous genetic background. Potentially all three genes, i.e., *FGA*, *FGB*, and *FGG* encoding for the alpha, beta and gamma chains, respectively, of the fibrinogen molecule may be affected and mutations spread along these genes. Patients with afibrinogenemia are homozygotes or compound heterozygotes, while individuals with hypofibrinogenemia are usually heterozygotes. Dysfibrinogenemia is usually associated with AD inheritance with two hotspots described, namely *FGA* p.Arg35 and *FGG* p.Arg301. These mutations are usually clinically benign, however, there are several other mutations which are associated with bleeding or thrombosis and predictive for the clinical phenotype, making genetic diagnosis beneficial [26,27,28].

Hereditary hemorrhagic telangiectasia (HHT) also known as Osler–Weber–Rendu disease is an AD multisystemic vascular disease with a worldwide prevalence of 1:5000–1:10,000 [29]. Statistics, however may underestimate the prevalence due to incomplete penetrance and underdiagnosis. In countries, like Hungary, where founder mutations exist, prevalence data may differ from the average value [30]. HHT is associated with abnormal blood vessel formation. Clinical diagnosis is based on clinical Curacao criteria including spontaneous and recurrent nosebleeds (with approximate frequency of 90–95%, multiple telangiectases at characteristic sites such as the lips, oral cavity, fingers and nose (90%); visceral lesions as gastrointestinal telangiectasia (20–80%) and pulmonary (30–50%), hepatic (32–48%) and cerebral or spinal (23%) arteriovenous malformation (AVM); and family history with a first-degree relative with HHT [31]. Approximately 85% of HHT cases have heterozygous family-specific mutations in the *ENG* or *ACVRL1* genes encoding endoglin and activin receptor-like kinase 1 (ALK1), members of the transforming growth factor-beta (TGF-β) superfamily. Rarely, mutations can be found in *MADH4* encoding SMAD4, a transcription factor acting as a mediator in the TGF-β /bone morphogenetic protein (BMP) pathway. Even more sparsely, mutations in *GDF2*, a gene encodes BMP, a ligand for ALK1, or in *RASA1* encoding RAS P21 protein activator 1 involved in cellular proliferation and differentiation, have been associated with HHT-like phenotype [32]. *RASA1* mutations result in the capillary malformation-arteriovenous malformation type 1 (CM-AVM1) syndrome overlapping with the symptoms of HHT [33]. Clinical and radiological features of HHT may be encountered with *RASA1* mutation [34]. As no laboratory method is available for HHT screening, in case of clinical suspicion molecular genetic tests represent first-line assays in the diagnostic procedure.

The aim of this study was to highlight certain differential diagnostic issues in congenital hemorrhagic disorders and to demonstrate the usefulness of molecular genetic investigations by summarizing the diagnostic work on our cases with vWD, HHT, fibrinogen disorders and with unexplained APTT prolongation. Being a tertiary care center with specialized hemostasis laboratory, we intended to ensure a comprehensive diagnostic care including genetic studies for patients with the aforementioned phenotypes and to re-evaluate cases with uncertain diagnosis by means of genetic investigations.

## 2. Materials and Methods

### 2.1. Patients

Patients were recruited during a 10-years period from the Clinical Center of the University of Debrecen. All patients went through comprehensive hemostasis laboratory investigations and plasma and DNA samples were reserved for further studies. This manuscript involves only those patients, in whom performance of genetic investigations was indicated due to uncertainty of diagnosis or if confirmation of initial diagnosis was required by genetic testing. Based on clinical and/or laboratory suspicion (see below) of vWD *n* = 63, hypo-or dysfibrinogenemia *n* = 27, HHT *n* = 10 and unexplained APTT prolongation *n* = 1 individuals were selected for NGS genetic analysis. Among patients with the clinical criteria of HHT only those without mutations in the *ENG* or *ACVRL1* genes are reported here. Informed consent for genetic screening was obtained from all subjects involved in the study.

### 2.2. Methods

#### 2.2.1. Routine Hemostasis Laboratory Investigations

Fasting blood samples were collected into 0.109 mol/L citrated vacutainer tubes (Beckton Dickinson, Franklin Lakes, NJ, USA) from all patients who underwent routine hemostasis laboratory investigations. Plasma samples were prepared by centrifugation at 1500 g for 20 min at room temperature and investigated immediately or stored at −80 °C until use. Screening tests of coagulation were performed on a Siemens BCS-XP coagulometer (Siemens, Marburg, Germany); Innovin and Pathromtin SL were used for PT and APTT determination, respectively (Siemens), and Labexpert Thrombin time and fibrinogen reagents were used for TT and Clauss-fibrinogen determination, respectively (Labexpert, Debrecen, Hungary). Fibrinogen antigen was determined by Siemens Immunonephelometry method on BN-Prospec analyser. Clotting factor activities were measured by one-stage clotting assay using Siemens deficient plasmas, except for prekallikrein and HMWK measurements, where Technoclone-deficient plasmas were used (Technoclone, Vienna, Austria). Laboratory diagnosis of vWD was performed according to algorithm based on recent recommendations [13,21]. vWF:Ag (BC von Willebrand reagent, immunonephelometry) and vWF:GPIbM (Innovance VWF Ac) were determined by Siemens reagents on the BCS-XP. vWF:CB was performed using type III collagen by Technoclone Technozym vWF:CBA ELISA kit, vWF:FVIIIB was performed by Asserachrom VWF:FVIIIB kit (Diagnostica Stago, Asnieres, France). Multimeric analysis of vWF was executed by an in-house immunoblotting method using Horseradish Peroxidase (HRP) -conjugated polyclonal rabbit anti-human vWF antibody (Dako, Glostrup, Denmark). Ristocetin induced platelet aggregation (RIPA) was executed in platelet-rich plasma obtained by centrifugation of citrated whole blood at 150 g, 15 min at room temperature, in the presence of 0.6 mg/L–1.5 mg/L ristocetin purchased from Helena (Helena Laboratories, Beaumont, TX, USA). Reptilase time was measured by reagent from Stago on ST4 mechanical coagulometer.

#### 2.2.2. Isolation of Genomic DNA

Genomic DNA was manually isolated from peripheral citrated whole blood by using the QIAamp DNA Blood Mini kit (Qiagen, Hilden, Germany). The isolations were carried out according to the manufacturer’s protocol. The DNA concentration was measured in the Qubit™ dsDNA HS Assay Kit using a Qubit™ 4.0 Fluorometer (Thermo Fisher Scientific, Waltham, MA, USA).

#### 2.2.3. Next-Generation Sequencing Method Using Illumina Platform

The input DNA was around 10 ng for each reaction. Libraries were created by a QIAseq targeted DNA custom panel (CDHS-15414Z-675, Qiagen, Hilden, Germany), according to manufacturer’s protocol. This panel covers 14 genes (*ACVRL1*, *ENG*, *MADH4*, *GDF2*, *RASA1*, *F5*, *F8*, *FGA*, *FGB*, *FGG*, *KLKB1*, *ADAMTS13*, *GP1BA* and *VWF*; ROI size: 48,149 bp). *ACVRL1, ENG, MADH4, GDF2* and *RASA1* were selected to resolve differential diagnosis in HHT phenotype; *FGA, FGB* and *FGG* were involved to differentiate among fibrinogen disorders; *F8, ADAMTS13, GP1BA* and *VWF* were selected for vWD diagnostics and finally, *F5* was added to the panel in order to screen for mutations/polymorphisms responsible for modifying patients’ phenotype (e.g., FV Leiden is very common in Hungary and may have a disease-modifying effect in bleeding disorders) [35].

The MiSeq System (MiSeq Reagent kit v2 300 cycles, Illumina, San Diego, CA, USA) was used for sequencing. A pooled sample of 12 patients with indexes were run simultaneously during a sequencing process. The libraries (final concentration of maximum 4 nM, pooled by equal molarity) were denatured by adding 0.2 nM NaOH and diluted to 20 pM with hybridization buffer (Illumina, San Diego, CA, USA). The final loading concentration of libraries was 8 pM. Sequencing was carried out according to the MiSeq instruction manual. Data were analyzed by NextGene software (SoftGenetics, State College, PA, USA). Coverage was at least 40× in all cases. A 20 bp intron region was examined before and after each exon. The first step was to convert the input data from FASTQ to FASTA format. Alignment was executed with version 37 of the Human genome (GRCh37). The identification of potential pathogenic variants was performed with the Human Gene Mutation Database (HGMD) database after screening the known polymorphisms (MAF > 0.01) described to date in the 1000 Genomes and dbSNP databases. The possible pathogenic mutations were validated by Sanger sequencing.

#### 2.2.4. Sanger Sequencing

The primers were designed using Primer3Plus software [36]. They are provided for readers upon request. The interested regions were detected by direct fluorescent sequencing on an ABI3130 Genetic Analyzer and analyzed by Sequencing Analysis 5.4 software (Thermo Fisher Scientific, Waltham, MA, USA).

#### 2.2.5. Genetic Data Analysis

The nomenclature used for amino acid numbering is according to the international recommendations for the description of sequence variants of the Human Genome Variation Society [37]. The variants found during the sequence analysis were compared with several databases. After screening for polymorphisms (dbSNP and 1000 Genomes databases), possible pathogenic variants were verified in HGMD [38], the ISTH-Scientific and Standardization Committee (SSC) VWF Online Database [39], the von Willebrand factor Variant Database [40], in the Associated Regional and University Pathologist (ARUP) Mutation Databases [41] and in the Human Fibrinogen Database [42].

#### 2.2.6. Prediction Methods

The pathogenicity of novel mutations was examined by Mutation Taster [43], PolyPhen2 [44], MutPred2 [45] and SIFT (sorting intolerant from tolerant) [46] prediction algorithms. Variants that identified were classified as pathogenic according to available databases in case of known mutations or according to the guidelines of the American College of Medical Genetics and Genomics [47].

## 3. Results

### 3.1. Genetic Diagnosis and Differential Diagnosis in von Willebrand Disease

#### 3.1.1. General Characteristics of von Willebrand Disease (vWD) Patients and Laboratory Results

We collected *n* = 59 patients with vWD, among which *n* = 34 index patients were registered, others were affected (*n* = 20) or unaffected (*n* = 3) family members (Table 1). Four patients were re-classified to hemophilia A based on the NGS results. Age at final diagnosis (date of confirmation by genetic testing) was highly variable in the patients; the youngest was 2 years old with type 2M deficiency and the oldest was 76 years old with 2N type. There was a female dominance in all types of the disease. In general, almost 80% of index patients had considerable bleeding tendency in their case histories and among them all patients with type 2M and 3 were bleeders. As expected, type 3 patients had the lowest levels of all vWF parameters, while in case of type 2B and 2N vWF:Ag was normal in most of the cases. vWF:Ac was the lowest in type 2A and 2M patients and it was normal in most cases with 2N and 2B. vWF:CB was an excellent marker of 2M and—as expected—it was normal in most cases with 2N and 2B. FVIII activity was decreased in 2N, however, it was also decreased in some cases with other functional defects of vWF. Decreased vWF:FVIIIB was abnormal in all investigated cases with 2N. Ratio of vWF:Ac to vWF:Ag was the lowest in 2A; however, due to low sample size, the difference among the different types was not statistically significant. As expected the FVIII:C to vWF:Ag ratio was the lowest in 2N. The multimeric analysis showed lack of HMWM in all cases with 2A, while some cases with 2B had normal multimeric pattern suggesting “atypical 2B vWD”. Other types showed no abnormal results in multimeric analysis.

#### 3.1.2. Classification of vWD According to Next-Generation Sequencing (NGS) Results

##### vWD Type 1 and 3 Patients

Four patients with vWF:Ag around 40% or lower showing quantitative vWD laboratory phenotype with mild symptoms or being symptomless were investigated by NGS testing (Table 2 and Supplementary Table S1). Altogether 6 different genetic variations were found in this group, among which 3 were novel ones (exon 6 c.657+2 T>C; exon 11 c.1187delT and c.1173_1183delAGGTCAATCAC). All, but one (p.Val1760Ile) variations considered as disease-causing based on available databases or mutation prediction, thus mild type 1 vWD diagnosis was established for these patients. Genetic investigation revealed no causative mutation in the fifth patient, but only a homozygous variation in exon 28 (p.Thr1381Ala). As this variation is considered as a likely benign functional polymorphism (rs216311) with the minor “A” allele frequency around 0.3, definitive diagnosis of vWD could not be established for her and “low vWF” was finally reported [48]. Type 3 vWD was diagnosed in three patients having very low vWF levels. One patient was homozygous for the c.2435delC (p.Pro812ArgfsTer31), while others were homozygous for the c.3379+1G>A splicing mutations.

##### vWD Type 2 Patients

Clinical and detailed laboratory characteristics of vWD type 2 patients are demonstrated in Supplementary Table S2. Among type 2 vWD, we have identified 6 unrelated individuals carrying 5 different known causative mutations considered as type 2A (Table 2). Four mutations affect the A2 domain (p.Ser1543Phe, p.Leu1503Pro, p.Arg1597Trp, p.Ile1628Thr) and one locates in D3 (p.Arg976Cys). Laboratory diagnosis of type 2A was unequivocally established in family 4 (Supplementary Table S2), where the ratio of vWF:Ac to vWF:Ag was clearly decreased below 0.6, and ratio of FVIII:C to vWF:Ag was around 1. RIPA was decreased even at high (1.5 mg/mL) concentration and lack of HMW multimers were detected in sodium dodecyl sulfate (SDS)-agarose gel analysis, however, we were not able to deeply analyse the triplet structure with certainty. Mutation found in this family is located at exon 28 (p.Ser1543Phe) affecting the A2 domain, a typical localization associated with the subtype IIA of vWD. Type 2A was also suggested by laboratory results in case of family 3 and patients 5 and 6 (Supplementary Table S2), and the genetic assay result (exon 28 p.Leu1503Pro, p.Arg1597Trp and p.Ile1628Thr, all A2 domain mutations) could also confirm the diagnosis and helped in sub-classification of 2A disease. Diagnosis of vWD 2A, however, was not as clear in patient 1, and family 2 before having the result of the genetic study. They shared the same mutation in exon 22 (p.Arg976Cys, D3 domain), which is considered as type 2A according to literature data. In the laboratory, ratio of vWF:Ac to vWF:Ag was around 1 and vWF:CB assay gave normal results. Instead, FVIII:C activity decreased in the two brothers of family 2, however it was normal in patient 1 and RIPA also showed normal result for her. It is notable, that in family 2 FVIII:C was decreased in both brothers, which first raised the possibility of having 2N vWD or mutation in *F8* simultaneously to vWF. Multimeric analysis was rather in accordance with type 2A, however, and genetic testing confirmed IIE subtype. No mutation was found in *F8*.

**Figure 1:**
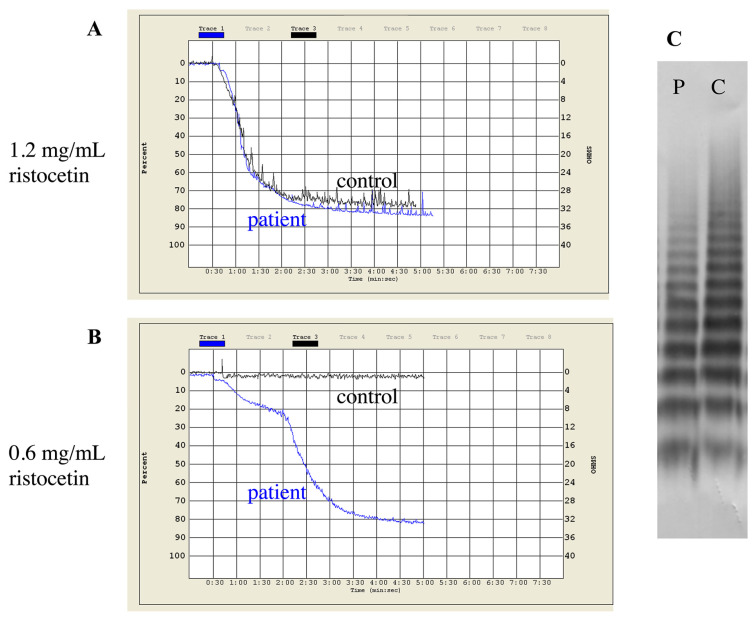
Patterns of ristocetin-induced platelet aggregation and multimer analysis of a type 2B vWD patient (Patient 10/1 in Supplementary Table S2). (**A**) Normal aggregation response to 1.2 mg/mL ristocetin. (**B**) Response to low dose of ristocetin (0.6 mg/mL). (**C**) Normal distribution of vWF multimers. vWF multimer analysis was performed by SDS-agarose gel electrophoresis. P = patient plasma, C = control plasma.

Type 2B vWD was indicated by genetic testing in case of seven unrelated patients (Table 2). Mutations were localized to the A1 and D3 domains of vWF. While A1 domain mutations lead to typical 2B laboratory picture with the lack of HMWM, other mutations may cause “atypical” 2B, that is more difficult to diagnose. A good example for this is the diagnosis of our four patients (patients 8/1, 9/1, 10/1, 11/1 in Supplementary Table S2), who were referred due to bleeding symptoms, while having normal screening tests of coagulation and platelet function. Platelet count was normal in all patients (between 155–283 G/L). vWF:Ag, vWF:Ac and FVIII:C were all normal or borderline and ratios of vWF:Ac to vWF:Ag and FVIII:C to vWF:Ag were around 1 in all cases. Multimeric analysis showed normal pattern and HMWM were present. The only abnormal finding was the enhanced platelet aggregation in the presence of low dose ristocetin (0.6 mg/mL), which raised the possibility of atypical 2B or platelet-type vWD (Figure 1). Since the mixing studies of ristocetin aggregation were inconclusive, the final diagnosis could be established on the basis of genetic test result. No mutation within *GP1BA* gene was detected, instead, we found a missense mutation within exon 28 of *VWF* (p.Pro1266Leu, D3 domain) in heterozygous form in all patients and affected family members. This mutation is listed in the HGMD database and considered as type 1 vWD. The ISTH vWD database, however suggests 2B and since direct evidence of enhanced platelet reactivity to ristocetin has been already published, we reported these cases as atypical 2B [49]. We detected typical 2B vWD with frequently described mutations in A1 domain in two patients with significant mucocutaneous bleedings (p.Arg1306Pro and p.Val1316Met).

Genetic testing helped in establishing final diagnosis in the case of a female patient with very low platelet count and a previous diagnosis of immune thrombocytopenic purpura (ITP) (patient 7/1 in Supplementary Table S2). A detailed clinical case-report is provided in Supplementary Materials 1. RIPA showed enhanced aggregation at low dose ristocetin, which was repeatable in the presence of control platelets and the patient’s plasma suggesting vWD 2B. NGS analysis did not find mutations within *GP1BA* and *ADAMTS13* genes but two mutations were detected, both in heterozygous form in *VWF*. One was located in the A1 domain in exon 28 (c.4381G>C, p.Ala1461Pro), typical for 2B, other was found in D2 domain (exon 15 c.1781C>G, p.Ala594Gly). The former is a novel mutation with a conclusion of being a pathogenic variant and the latter is a known mutation associated with type 1 vWD according to databases (Table 3). We cannot justify whether the two mutations locate in cis- or trans-positions due to lack of samples from family members (parents of the index patient have already passed away and there are no siblings).

Type 2M vWD was diagnosed in six unrelated patients with clinically moderate or severe bleeding phenotype (Table 2, Supplementary Table S2). vWF:Ag, vWF:Ac, vWF:CB and FVIII:C were all low resulting in ratios above the cut off values of 0.6, multimeric pattern was normal suggesting a quantitative vWD. Based on genetic testing vWD Vicenza was diagnosed (exon 27, D3 domain, p.Arg1205His), which was first described in two Italian kindreds by Mannucci et al. [50]. This variant is also considered as quantitative disease with enhanced clearance of vWF. A boy with bleeding after surgery was referred to our laboratory (19/1 in Table S2), where very low vWF:Ac to vWF:Ag ratio (0.3) was found besides low vWF:Ag (17%) and RIPA. Multimeric distribution was normal and NGS testing revealed two mutations in heterozygous forms, one is located in the A1 domain considered as 2M type (p.Leu1296Pro) according to the ISTH database, and the other mutation is a novel one located in the CK domain (p.Asp2717Asn), which is considered as disease-causing according to only one prediction tool, while others predicted as benign (Table 3). We cannot confirm whether the two mutations are in cis -or trans-positions, since no relatives are available for testing, however the presence of the p.Leu1296Pro is enough to diagnose the patient as 2M vWD.

**Figure 2:**
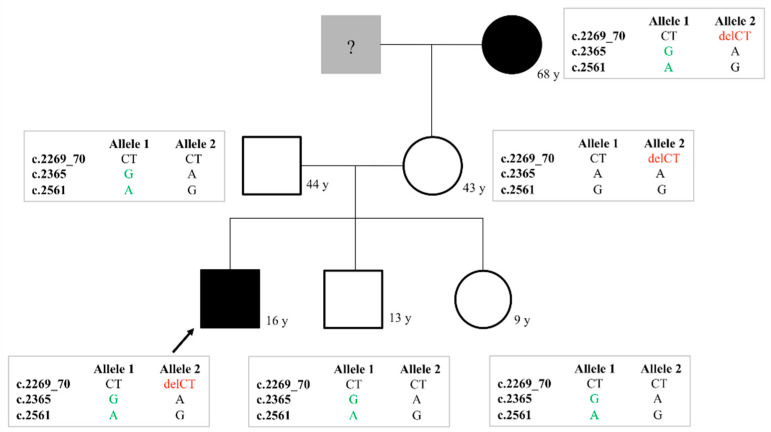
Family chart of a type 2N vWD family (Family 21 in Supplementary Table S2). The variants that occur in the family are c.2269_70delCT (p.Leu757Valfs*22) in exon 17; c.2365A>G (p.Thr789Ala) in exon 18 and c.2561G>A (p.Arg854Gln) in exon 20.

Eleven families were investigated due to remarkably low FVIII:C, where the final diagnosis of vWD and/or the differential diagnosis of vWD 2N and hemophilia A was performed by the help of genetic studies (Table 2, Supplementary Table S2). The most frequent mutation (found in 6 unrelated families) in this cohort was the p.Arg854Gln D’ domain mutation either in homozygous or in compound heterozygous form. The benign variant p.Thr789Ala was detected in four families [48]. Homozygous p.Arg854Gln was demonstrated as the cause of the AR type 2N vWD in two families (families 24 and 25 in Supplementary Table S2). The p.Arg854Gln mutation was combined with others in four families. The combination of the p.Arg854Gln with different quantitative vWD mutations (p.Arg1779Term, c.3379+1G>A) was also found. The p.Arg854Gln and c.3379+1G>A are suggested to locate in trans-position, based on results of the family study. The p.Arg854Gln was combined with p.Tyr795Cys in trans-position in a young girl with 19% FVIII:C and 4% vWF:FVIIIB (23/1 in Supplementary Table S2). A family with 6 members was referred to us due to bleeding following surgery in the index patient, a 16 years old boy (21/1 in Supplementary Table S2) (Figure 2). He was compound heterozygote for the 2N p.Arg854Gln and a type 3 mutation (p.Leu757Valfs*22) and carrier of the benign p.Thr789Ala variant. The p.Arg854Gln was in cis-position with p.Thr789Ala. His brother and sister with normal FVIII:C and vWF levels inherited this allele, which came from their father, while the type 3 allele was inherited from the mother leading to moderately and proportionally low vWF:Ag, vWF:Ac and vWF:CB and disproportionally low FVIII:C and low vWF:FVIIIB. Interestingly, the maternal grandmother showed the same genotype as the index patient with the same clinical and laboratory phenotype.

Hemophilia A carriership was revealed by NGS testing in two females with significant bleeding in their case histories and hemophilia A was diagnosed in two males with 3% and 4% FVIII:C (Supplementary Table S2).

### 3.2. Genetic Diagnosis in Fibrinogen Abnormalities

#### 3.2.1. Mutations Associated with Dysfibrinogenemia Phenotype

##### Mutations Affecting FGA p.Arg35 Position

Among patients suspected for fibrinogen abnormality five families were registered with mutations affecting FGA p.Arg35 position (Table 4 and Supplementary Table S3). In one patient p.Arg35Cys was found in heterozygous form, while four families were found to be carriers of the p.Arg35His mutation. In all families the index patients were referred due to prolonged clotting times at preoperative screening without clinical symptoms. There was a family with simultaneous presence of antithrombin (AT) deficiency, a severe thrombophilia [51]. Clinical details are described in the Supplementary Materials 1. Although TT was sensitive enough to detect fibrinogen abnormality with FGA p.Arg35 mutation, RT proved even more sensitive, as all carriers of mutations at the FGA p.Arg35 position showed indefinitely prolonged RT. Mean ratio of fibrinogen measured by the Clauss method to fibrinogen antigen was 0.24 ± 0.08 in FGA p.Arg35 mutations, which was the best predictor of the presence of causative mutations. A female patient (5/1 in Supplementary Table S3) was a double carrier of the FGA p.Arg35His and p.Thr331Ala mutations. As no family member was available for testing, we do not know, if the two FGA mutations are located in cis- or trans positions, however, p.Thr331Ala is suggested not being disease-causing according to different prediction tools and databases, it is rather considered as polymorphism [52].

##### Mutations Affecting Other Positions in Fibrinogen Genes

Three families were registered with the other frequent, the *FGG* p.Arg301His mutation (Table 4). A mother and daughter (6/1 and 6/2 in Supplementary Table S3) were found to be heterozygous carriers of the *FGA* p.Arg38Ser mutation, and one patient (7/1 in Supplementary Table S3) was registered with the *FGB* p.Arg196Cys mutation. *FGA* p.Arg38Ser was associated with bleeding according to available databases, our patients, however, have had no symptoms, so far. Patients carrying the *FGG* p.Arg301His mutation had mild or moderate bleeding symptoms, or remained asymptomatic until the date of manuscript preparation. The patient with *FGB* p.Arg196Cys mutation was a heavy bleeder. In the laboratory, *FGA* p.Arg38Ser mutation associated with prolonged TT and RT. In the case of *FGG* p.Arg301His only moderate TT prolongation could draw the attention to potential fibrinogen disorder, the prolongation of RT was more pronounced than that of TT; however, it remained below 100 s. Functional fibrinogen levels and the ratio of Clauss-fibrinogen to fibrinogen antigen (0.48 ± 0.23) were higher than in the case of *FGA* p.Arg35 mutations. Screening tests of coagulation including RT were not suspect for any coagulation abnormality in the case of *FGB* p.Arg196Cys mutation and they were normal on both optical and mechanical coagulometers, only the coagulation curve obtained from the optical coagulometer showed some deviation from normal. Clauss-fibrinogen was low and a discrepancy of this and fibrinogen antigen was demonstrated, which suggested fibrinogen abnormality. Unexpectedly, upon NGS screening in the patient carrying *FGB* p.Arg196Cys mutation (patient 7/1 in Supplementary Table S3) a novel variant within *VWF* was also found. Mutations, described at the same position, so far, were associated with type 3 disease and it was suggested associate with type 1 in heterozygous form [53,54]. *VWF* p.Arg34 is completely conserved across species, and the p.Arg34Gln is predicted to have a detrimental effect on vWF. Based on the NGS results the diagnosis of combined coagulopathy (dysfibrinogenemia and vWD) was reported.

A novel mutation (*FGG* p.Met362Lys), associated with dysfibrinogenemia laboratory phenotype was found in a male patient suffering from thrombotic symptoms and with positive family history for deep venous thrombosis (patient 14/1 in Supplementary Table S3). In our laboratory, TT and RT showed slight prolongation, fibrinogen level was disproportionally decreased, corresponding to dysfibrinogenemia. The mutation locates at a typical region, close to the C-terminal end of the fibrinogen gamma chain and it is disease-causing according to all prediction tools (Table 3).

#### 3.2.2. Mutations Associated with Hypofibrinogenemia Phenotype

**Figure 3:**
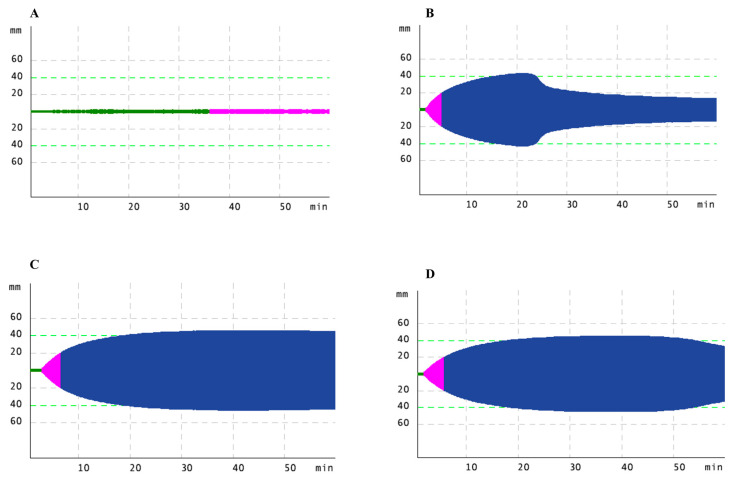
Rotational thromboelastometry (ROTEM) analysis in a patient with a novel FGB mutation (patient 13/1 in Supplementary Table S3). FIBTEM, EXTEM, INTEM and APTEM tests were performed detailed in the text. ROTEM curves for (**A**). FIBTEM, (**B**). EXTEM, (**C**). INTEM, (**D**). APTEM tests.

A known (*FGB* p.Tyr356Cys) and a novel mutation (*FGB* p.Trp474*) were found in the background of hypofibrinogenemia laboratory phenotype. Family with the *FGB* p.Tyr356Cys mutation (family 11 in Supplementary Table S3) was also carrier of different functional polymorphisms within *F7* and *F5* genes, complicating their laboratory phenotype and diagnosis. For detailed information, please see Supplementary Materials 1. Clinical data of patient carrying the novel *FGB* p.Trp474* is also summarized in Supplementary Materials 1. The C-terminal part of Bβ-chain is a highly conserved globular structure, which practically do not tolerate mutations and it was predicted to be disease-causing by MutationTaster [28]. A global assay, rotational thromboelastometry (ROTEM), was undertaken to investigate functional aspects of the blood clot in the patient (Figure 3). Clotting times (CT) and clot formation times (CFT) reflecting to the speed of solid clot formation were prolonged in all settings (EXTEM, INTEM, FIBTEM and APTEM) and the α-angle was markedly lower. Maximal clot firmness, which is generally used to assess fibrin clot strength, was low in all assays. The clot seemed to be sensitive to lysis in EXTEM (and in FIBTEM) assays. These findings confirm hypofibrinogenemia with a bleeding phenotype associated with this novel mutation; however, more detailed biochemical studies are required to explore its consequences at a molecular level.

#### 3.2.3. Mutations Associated with Other (Non-Hemostasis Related) Phenotype

Two related patients (12/1 and 12/2 in Supplementary Table S3) were identified with the *FGA* p.Glu545Val mutation. Both suffered from renal amyloidosis, a known complication associated with this genotype.

### 3.3. Genetic Testing in Hereditary Hemorrhagic Telangiectasia Phenotype

**Figure 4:**
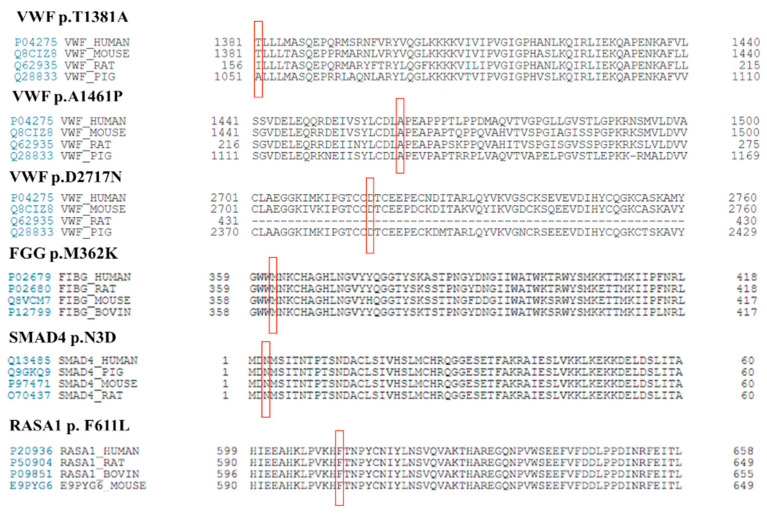
Homology analysis of novel missense mutations. Affected amino acids are indicated in red squares.

Patients with *ENG* and *ACVRL1* mutations are described elsewhere or subjects of another paper dealing with clinical aspects of HHT [55,56]. Only two probands were negative for both genes, 64-year-old and 62-year-old female patients referred due to recurrent heavy nose bleedings requiring interventions for 6 years and 7 years, respectively. The former patient was labeled as definitive HHT based on 3 positive Curacao criteria out of the 4, as recurrent epistaxis, telangiectases at lips and a positive family history. In her case, a novel, likely pathogenic *MADH4* missense mutation (c.7A>G, p.Asn3Asp) was detected in heterozygous form. The second patient had a negative family anamnesis and no mucocutaneous telangiectasia was found by physical examination, moreover, nasopharyngoscopy, nasal sinus CT and cranial CT angiography were all negative for AVM (1 positive Curacao criterion). All hemostasis investigations including coagulation and platelet functional studies showed normal results. NGS analysis detected a novel *RASA1* variant (c.1833T>G, p.Phe611Leu) in heterozygous state (Table 3). Both mutations locate at a conserved region of their genes, and the mutations affect conservative amino acids according to the results of homology investigations (Figure 4). Genetic counselling and family screening were advised in both cases.

### 3.4. Exploring the Background of Unexplained Activated Partial Thromboplastin Time (APTT) Prolongation

An 11-year-old girl with no bleeding in her history was screened for coagulation disorders as a routine procedure before tonsillectomy. Among coagulation screening tests APTT showed an isolated, extreme (over 200 s) prolongation, which was corrected by the 1:1 mixing with normal plasma (APTT of patient:control 1:1 mix 30.2 s, APTT of control 28.2 s). PT and TT were normal (8.2 s and 18.1 s, respectively). Fibrinogen was within the reference interval, 2.88 g/L. The presence of lupus anticoagulant was excluded based on the correctable APTT, that remained correctable even after 1 h incubation at 37 °C, on the corrigable prolongation of lupus anticoagulant sensitive APTT (APTT-LA), which was 136.7 s for the patient and it was 34.6 s in the 1:1 mixing study (APTT-LA of control plasma was 33.0 s) and on the negative result of diluted prothrombin time. By investigating intrinsic clotting factor activities no abnormal results were observed (activities of FVIII, FIX, FXI and FXII were 61%, 95%, 94% and 79%, respectively). The reference interval is 60–150% in case of all factors. As this laboratory phenotype is known to be characteristic even for contact phase factor deficiencies, we performed PK and HMWK determinations, as well. While HMWK was within the corresponding reference interval (71%), the activity of PK was undetectably low (below 1%). Genetic investigation (*KLKB1*) of the patient and her parents was performed in order to confirm the diagnosis of hereditary PK deficiency (to exclude acquired PK deficiency) and a compound heterozygote state was found. The patient was heterozygous for a known causative missense mutation (c.1643G>A, p.Cys548Tyr) also found in her mother and for a novel nonsense one (c.406G>T, p.Glu136Ter) detected also in her father [57]. Pathogenicity of the novel mutation was tested by the prediction tool MutationTaster and it was found to be “disease causing”. No mutation was found in the proband’s *F8* and *VWF* genes excluding her carrier status in hemophilia A or in vWD. By clarifying the background of APTT prolongation we could reassure the operation team about the benign nature of this laboratory abnormality.

## 4. Discussion

In this paper we present *n* = 63 individuals investigated for vWD, *n* = 27 patients with fibrinogen abnormalities, *n* = 2 patients with HHT phenotype and *n* = 1 individual with indefinitely prolonged APTT. We performed the comprehensive hemostasis laboratory investigations and used the NGS method to clarify the diagnosis and to resolve differential diagnostic problems. Altogether we identified 47 mutations, *n* = 29 mutations (6 novel) in vWD, *n* = 4 mutations leading to hemophilia A, *n* = 10 mutations (2 novel) in fibrinogen disorders, two novel mutations in HHT-like phenotype and two mutations (1 novel) in PK deficiency.

In vWD, genetic testing has a significant role especially in patients with type 2 and 3 diseases [58,59]. In type 2 vWD there are typical *VWF* regions, where mutations cause well-defined phenotypes, like exon 28 mutations, which are responsible for 2A, 2B and 2M, mutations in exons 11–16, 24–26 and 51–52 leading to 2A, exon 18–25 mutations associate with 2N phenotype. In the cases of type 1 and 3 vWD, mutations can spread along *VWF,* complicating classical molecular genetic approach. Moreover, in case of simultaneous occurrence of two functional mutations or a functional one and a quantitative variation genotype-phenotype associations are more difficult to describe.

Genetic analysis of vWF helps to distinguish vWD type 1 from the so-called “low-vWF” without having mutation within *VWF* gene. We have identified five pathogenic or likely-pathogenic novel mutations in *VWF* in heterozygous form and could interpret those patients as mild type 1 vWD. The p.Thr1381Ala polymorphism, that is found in homozygous form in a patient with slightly low vWF levels is considered as a benign variation. Homozygosity for the threonine allele associates with a higher affinity to platelet GPIbα receptor at least in static conditions in vitro, our patient, however was homozygous for the alanine allele, which further suggests that presence of this genetic variation has no clinical significance [60].

ISTH suggests to sub-divide type 2A vWD into IIA, IIC, IID and IIE [3,61] based on the different molecular pathomechanism and mutations in different domains. In experienced hands, SDS-agarose gel electrophoresis at low-resolution is able to discriminate among these sub-types, however, most of the laboratories can perform this method only with uncertainty. A commercially available semi-automated method, HYDRAGEL VW multimer assay has been recently introduced in order to simplify and standardize vWF multimeric analysis. This assay, however, is used only at a pre-defined resolution, and therefore it is not able to classify type 2A into its sub-classes [18]. Genetic analysis can help in this issue by identifying the causative mutation in the characteristic domains of 2A subtypes. In our patients four out of five mutations were found in the A2 domain leading to IIA subtype, while one mutation in the D3 domain associated with IIE.

Type 2B is associated with an abnormal multimeric structure in most, but not all cases and with an enhanced RIPA even at a low dose ristocetin [18,62]. In case of type 2B vWD differential diagnosis of platelet-type vWD and the “atypical” 2B and Bernard–Soulier syndrome or sometimes other diseases with low platelet count may cause difficulties [21]. Genetic analysis of *VWF* and *GP1BA* can help in this issue, like in our cases with the causative mutation in the D3 domain leading to normal multimeric pattern, despite enhanced RIPA. Here “atypical” 2B could be diagnosed. In the case of our patient with ITP misdiagnosis genetic analysis helped to clarify the situation and classified the patient as vWD. The novel mutation found in her case (p.Ala1461Pro) was predicted as disease causing according to the majority of prediction tools.

Type 2M is often misdiagnosed as 2A or type 1, moreover, according to the site of mutation it can be associated with a collagen-binding defect (mutations in the A3 domain, or in some cases in the A1 domain), or with a reduced platelet-dependent activity (mutations in the A1 domain) [63]. Classification of vWD Vicenza (p.Arg1205His) has always been controversial [64]. In the laboratory no discrepancy between vWF:Ac and vWF:Ag is found, enhanced clearance of vWF is suggested in some studies, which findings are rather in accordance with a type 1 variant. In multimeric pattern analysis presence of ultra-large multimers was observed in the early studies, suggesting rather type 2 disease; others, however did not find any abnormality in multimeric pattern in families carrying the same mutation. vWD Vicenza is considered now as a dominant vWD type 1 qualitative defect with normal secretion but rapid clearance of vWF with equally low levels of FVIII:C, vWF:Ag, vWF:RCo, vWF:CB and the presence of unusually large vWF multimers in the plasma [65]. Whether being a type 1 or 2M disease it is more important that the vWD Vicenza variant has an impact on 1-deamino-8-D-arginine vasopressin (DDAVP) administration due to its excellent but short-lived effect, highlighting the usefulness of establishing genetic diagnosis in this type of disease.

Differentiation of type 2N vWD (AR) from hemophilia A (XR) may also be challenging in certain situations, and if it is combined with quantitative *VWF* mutation, the situation can be even more problematic [20,66]. Activity of FVIII (FVIII:C) shows a low value in hemophilia A, sometimes in carriers also, and the ratio of FVIII:C to vWF:Ag is below the cut off value (i.e., below 0.6) in these individuals, similarly to that of vWD 2N patients [59]. Performance of the vWF:FVIIIB assay is able to discriminate in most of the cases, however, confirming the laboratory diagnosis by genetic testing may be useful [21]. In our cases the p.Arg854Gln mutation was the most common either in homozygous or in compound heterozygous form. It is worth commenting that this mutation is often combined with other 2N mutations, or with mutations associated with other types, leading to a combined phenotype of 2N and type 1, which makes laboratory results equivocal [20]. In our cohort, combinations of two 2N mutations and a 2N with type 1 or type 3 variants were also detected. Four patients were re-classified to hemophilia A after genetic testing.

Concerning fibrinogen abnormalities, most of our patients were carriers of the frequent mutations in *FGA* exon 2 (p.Arg35Cys and p.Arg35His) or in *FGG* exon 8 (p.Arg301His), which are responsible for more than 80% of all variants in dysfibrinogenemia [67]. Our cases with *FGA* p.Arg35 and *FGG* p.Arg301 mutations demonstrate well the clinically benign nature of this type. All carriers were free of symptoms, or had mild bleedings. Thrombosis was not observed in these patients. Carriers of mutation (p.Arg38Ser) affecting the neighboring region were also symptomless. Mutations at these regions may affect fibrin polymerization or lead to fibrinolysis resistance according to biochemical studies [68,69].

Laboratories, where performance of TT and RT is not part of routine coagulation screening, may misdiagnose certain types of fibrinogen abnormalities, like the dysfibrinogenemia caused by *FGA* p.Arg35Cys/His and p.Arg38Ser mutations, or the *FGG* p.Arg301His mutation. If PT and APTT reagents are not sensitive for fibrinogen abnormalities, like in our laboratory, they may detect only the most severe fibrinogen disorders, although symptoms may develop in less severe cases, as well. We have demonstrated that in case of *FGB* p.Arg196Cys even TT and RT were not able to predict a fibrinogen disorder, only careful analysis of an abnormal coagulation curve on an optical coagulometer suggested the presence of a coagulation defect. Clauss-fibrinogen, however, was a good marker of dysfibrinogenemia in this case [70]. If fibrinogen is only moderately decreased and remains above 1 g/L (corresponding to about 30% of normal fibrinogen level) coagulation screening tests including TT are not expected to be prolonged, therefore, fibrinogen disorders—despite having causative mutations and clinical symptoms—may remain undiagnosed if based on normal screening test results no fibrinogen measurement is performed.

Moreover, both coagulation screening test results and fibrinogen levels may fall within the reference intervals, despite the presence of genetic alterations. A good example for this is the family with the *FGA* p.Glu545Val mutation, which resulted in renal amyloidosis. Hereditary fibrinogen alpha chain amyloidosis is a rare autosomal dominant inherited disorder, which is the most prevalent form of hereditary renal amyloidosis. The mutated fibrinogen alpha chain can accumulate in the kidneys forming amyloid fibrils, which leads to organ dysfunction and can be fatal [71]. The most common variant associated with fibrinogen amyloidosis is *FGA* p.Glu545Val. Genotyping of fibrinogen-amyloidosis patients is also important from the point of view of future perspectives. Kidney transplantation seems to be a viable option for patients with the *FGA* p.Glu545Val mutation [72].

In congenital fibrinogen deficiency clinical phenotype is highly variable and may be influenced not only by mutations within *FGA, FGB* or *FGG* genes, but also other hemostasis conditions may have a contribution [67]. We can demonstrate some examples for this in our cohort. Clinical phenotype was shifted to thrombotic direction in the family carrying both dysfibrinogenemia variant and antithrombin deficiency and in the patient who was double carrier of dysfibrinogenemia and FV Leiden mutation. Carriership of a likely pathogenic mutation in the *VWF* gene might act as an additive effect to *FGB* p.Arg196Cys mutation to result in a bleeding phenotype. The son of the proband, carrying the *FGB* mutation but not the *VWF* mutation, has been free of bleeding. The most complex genotype-phenotype relationship in our patient group is probably seen in the family carrying simultaneously *FGB, F5* and *F7* mutations. The *FGB* p.Tyr356Cys found in this family was described with bleeding phenotype in one family earlier [73], which corresponds to the symptoms of two out of the four family members in our group. An explanation to the borderline factor VII levels was found by genetic investigation, as three common variants (F7 c.−323 ins10; c. −122T>C; c.1241 G>A, p.Arg413Gln) were detected in heterozygous form in all family members. Two of these mutations, which are located upstream the initiation codon of F7, were found to associate with decreased promoter activity, thus lower expression level of factor VII [74,75]. The p.Arg413Gln (rs6046) mutation is also common (global minor allele frequency 0.14) and it may also contribute to lower factor VII levels [76]. Besides having an explanation to factor VII levels in the family, the more important question is whether these variations may have a considerable contribution to the clinical phenotype and whether they may have a predictive value. Based on previous reports we suggest that *F7* mutations behave at least as minor additive factors to shift the hemostasis balance towards an anti-coagulation effect [77,78,79,80,81,82]. The NGS study revealed three mutations within the *F5* gene in the index patient. Neither of them associates with factor V deficiency. The p.Lys858Arg (rs4524) is a common polymorphism (MAF 0.27), which was found to increase the risk of venous thrombosis slightly in a number of studies including a large meta-analysis [83,84]. More studies, however, are needed to uncover whether this polymorphism itself is responsible for the slight pro-thrombotic effect [85]. The p.Met1764Val (rs6030) has a minor allele frequency of 0.3. One study found increased thrombosis risk in carriers of rs6030 minor allele [86]. Variation p.Met2148Thr (rs9332701) is rare with MAF 0.03. This variant was shown to be associated with low factor V level without pronounced effect on clinical phenotype [87]. In summary, we do not consider the detected *F5* variants likely to represent further bleeding risk and their pro-thrombotic effects, if any, are subtle.

Based on clinical (Curacao) criteria, diagnosis of HHT certainly does not require genetic testing [88]. As no laboratory assay is available to confirm the diagnosis, genetic testing has a significant role. A significance of genetic testing is the confirmation or exclusion of HHT in young asymptomatic individuals in families with pathogenic mutations. Identification of founder effects might simplify the genetic diagnosis of new HHT patients from the corresponding geographic region [56]. Another goal of genetic testing is to find mutations in genes rarely associated with HHT, like in *MADH4* in our case to support the clinical diagnosis. Genetic testing of patients showing symptoms similar to HHT is also important to clarify the background of the clinical findings. If only *ENG* and *ACVRL1* genes are investigated diagnosis of HHT or capillary malformation-arteriovenous malformation syndrome (HHT-like syndrome) may be delayed or they may remain undiagnosed. Use of NGS-based gene panels including not only *ENG, ACVRL1* and *MADH4* but also *RASA1* and *EPHB4* genes, which are associated with the HHT-like syndrome accelerates the diagnosis of these hereditary vascular disorders [32]. There are specific clinical features associated with one or other gene mutation. Mutations within *ACVRL1* and *MADH4* may be associated with pulmonary hypertension, while basal cell carcinoma was described in *RASA1* mutations. In the case of novel mutations within genes related to the HHT or HHT-like phenotype, it is important to describe genotype–phenotype relations to help others predict the age, type and severity of bleedings. Novel treatment modalities made genetic confirmation of HHT even more important [89,90].

The background of an alarmingly prolonged coagulation screening test must always be clarified before invasive intervention. In our case the reason for having an extremely prolonged APTT was clarified by finding two mutations in the *KLKB1* gene [8]. Using NGS technology we were able to establish the diagnosis of inherited PK deficiency rapidly and could avoid a significant delay in undertaking a tonsillectomy. Demonstrating the benign nature of APTT prolongation is of great importance from the point of view of further patient management, since this laboratory phenomenon will result in frustration and potential delay in any interventions.

## 5. Conclusions

It is obvious that genetic testing provides a higher-level evidence for diagnosing bleeding disorders. However, care should be taken upon describing a genetic variant in hemorrhagic diseases. In the case of novel variants clinical and laboratory phenotype should be clearly connected to the genotype before considering it as pathogenic. As mutation prediction tools are not always concordant and sometimes have opposite judgement of pathogenicity, in vitro biochemical studies may be necessary to determine the significance of the mutation [91]. Moreover, if previous information does not match with present findings, re-classification of already described mutations may be necessary. In order to upgrade the different bleeding disorders databases and to clarify the consequences of the mutations, well-described cases in terms of clinical and laboratory phenotypes using standardized, advanced laboratory methods and algorithms are useful. When genetic databases are reliable and helpful in bleeding risk prediction and patient management, diagnostic procedures will be simplified bypassing several time-consuming and laborious procedures [21]. Biochemical assays, however, will always be inevitable either in the case of novel genetic variants or as initial diagnostic steps.

## Figures and Tables

**Table 1:** Clinical and laboratory characteristics of von Willebrand disease patients. Patients with hemophilia A (*n* = 4) are not represented in the table. Unaffected family members (*n* = 3) are not described in the table. Reference intervals for von Willebrand antigen (vWF:Ag), von Willebrand activity (vWF:Ac), von Willebrand factor collagen binding capacity (vWF:CB), activity of coagulation FVIII (FVIII:C) and FVIII binding of von Willebrand factor (vWF:FVIIIB) are 50–160%, 61–179%, 60–130%, 60–150% and >40%, respectively. * epistaxis, bleeding following tooth extraction/trauma/surgery, easy bruising, menorrhagia, post-partum hemorrhage, umbilical bleeding, gastrointestinal bleeding, retinal bleeding, gum bleeding, muscle hematoma, undefined bleeding. ND, no data. § Current age and age at genetic diagnosis was significantly higher in 2M probands as compared to type 1, 2B and 2N probands. vWF:Ag and vWF:Ac were significantly lower for type 3 probands as compared to 2B and 2N and for type 2M probands as compared to 2B and 2N. vWF:Ac was also significantly lower for type 2A probands as compared to 2N. FVIII:C was significantly lower in type 3, 2M and 2N probands as compared to type 1, 2A and 2B. FVIII:C/vWF:Ag ratio was significantly lower in 2N probands as compared to type 1, 2A and 2B.

	All Suspected vWD Patients *n* = 56	All vWD Probands *n* = 34	Type 1 *n* = 5	Type 2A *n* = 6	Type 2B *n* = 7	Type 2M *n* = 6	Type 2N *n* = 7	Type 3 *n* = 3	*p* Value §
Female/Male (%)	35/21 (65/35)	24/10 (71/29)	4/1 (80/20)	4/2 (67/33)	5/2 (71/29)	4/2 (67/33)	4/3 (57/43)	3/0 (100/0)	0.898
Median current age in years (range)	39 (4–78)	34 (4–77)	37 (29–45)	24 (13-44)	45 (21–75)	64 (4–76)	18 (15–77)	32 (32–55)	0.045
Median age at genetic diagnosis in years (range)	35 (2–76)	33 (2–75)	35 (27–43)	19 (11-42)	41 (13–73)	62 (2–74)	17 (10–75)	30 (30–53)	0.058
vWF:Ag (%)	42 (3–219)	39 (<3–125)	38 (32–42)	38 (12-77)	58 (25-125)	14 (7–88)	48 (16–96)	<3 (<3–20)	0.013
vWF:Ac (%)	34 (4–218)	29 (<4–107)	34 (29–46)	14 (7-70)	49 (13–104)	12 (5–20)	52 (17–107)	<4 (<4–<10)	0.005
vWF:CB (%)	51 (6–221)	30 (6–151)	33 (30–36)	20 (6-65)	58 (16-91)	12 (7–14)	61 (24–151)	ND	0.102
FVIII:C (%)	43 (1–148)	33 (<1–148)	63 (47–69)	44 (32-148)	76 (33–147)	18 (7–36)	18 (8–24)	<1 (<1–4)	<0.001
VWF:FVIIIB (%)	30 (4–123)	14 (<1–80)	ND	80	ND	ND	13 (<1–37)	ND	ND
vWF:Ac/vWF:Ag	0.85 (0.13-3.33)	0.91 (0.16–3.33)	0.91 (0.79–1.2)	0.54 (0.33–0.91)	0.84 (0.36-1.12)	1.01 (0.16–2.29)	1.05 (0.84–1.17)	1.33 (0.20–3.33)	0.146
vWF:CB/vWF:Ag	0.99 (0.16–1.71)	0.90 (0.16–1.71)	0.84 (0.79–0.9)	0.71 (0.50–0.84)	0.99 (0.34–1.21)	0.62 (0.16–1.71)	1.33 (0.98–1.57)	ND	0.239
FVIII:C/vWF:Ag	1.00 (0.2–3.7)	1.21 (0.05–3.70)	1.82 (1.12–1.9)	1.51 (0.42–3.70)	1.21 (0.74–1.81)	1.44 (0.20–2.12)	0.42 (0.20–0.57)	0.33 (0.05–1.33)	0.002
Ratio of patients with bleeding symptoms * (%)	35/54 (65.0%)	26/34 (76.5%)	3/5 (60.0%)	4/6 (66.7%)	6/7 (85.7%)	6/6 (100%)	4/7 (57.0%)	3/3 (100%)	0.306

**Table 2:** Detected mutations in von Willebrand disease.

vWD Type	No. Cases (No. Families)	Mutation Type	Domain	Exon	Mutation	
Type 1	1 (1)	splicing	D1	6	**c.657+2T>C**	-
	1 (1)	missense	A3	30	c.5278G>A	p.Val1760Ile
	1 (1)	small deletion	D2	11	**c.1187delT**	**p.F396Sfs**
	1 (1)	small deletion	D2	11	**c.1173_1183delAGGTCAATCAC**	**p.T391delLfs**
	1 (1)	missense	A2	28	c.4751A>G	p.Tyr1584Cys
	1 (1)	missense	D4	34	c.5768T>C	p.Leu1923Pro
	1 (1)	missense	A1	28	c.4141A>G	p.Thr1381Ala
	1 (1)	missense	D2	15	c.1781C>G	p.Ala594Gly§
	1 (1)	nonsense	A3	31	c.5335C>T	p.Arg1779*§§
Type 3	2 (1)	small deletion	D’	18	c.2435delC	p.Pro812Argfs*31
	3 (3)	splicing	D3	25	c.3379+1G>A§§	-
	3 (1)	small deletion	D2	17	c.2269_70delCT	p.Leu757Valfs*22§§
Type 2A	3 (2)	missense	D3	22	c.2926C>T	p.Arg976Cys
	3 (1)	missense	A2	28	c.4508T>C	p.Leu1503Pro
	4 (1)	missense	A2	28	c.4628C>T	p.Ser1543Phe
	1 (1)	missense	A2	28	c.4789C>T	p.Arg1597Trp
	1 (1)	missense	A2	28	c.4883T>C	p.Ile1628Thr
Type 2B	1 (1)	missense	A1	28	**c.4381G>C**	**p.Ala1461Pro**
	5 (4)	missense	D3	28	c.3797C>T	p.Pro1266Leu
	1 (1)	missense	A1	28	c.3917G>C	p.Arg1306Pro
	1 (1)	missense	A1	28	c.3946G>A	p.Val1316Met
Type 2M	6 (5)	missense	D3	27	c.3614G>A	p.Arg1205His#
	1 (1)	missense	A1	28	c.3887T>C	p.Leu1296Pro
	1 (1)	missense	CK	50	**c.8149G>A**	**p.Asp2717Asn**
Type 2N	10 (4)	missense	D’	18	c.2365A>G	p.Thr789Ala
	4 (1)	missense	D’	18	c.2384A>G	p.Tyr795Cys
	13 (6)	missense	D’	20	c.2561G>A	p.Arg854Gln
	1 (1)	missense	D3	21	c.2771G>A	p.Arg924Gln

Novel mutations are indicated in Bold. p.Thr1381Ala variant is considered as likely benign functional polymorphism. p.Thr789Ala is considered as benign variant. § This mutation was combined with p.Ala1461Pro in a patient showing 2B phenotype. §§ These mutations were combined with the 2N p.Arg854Gln mutation. #vWD Vicenza is also considered as type I vWD.

**Table 3:** Mutation pathogenicities as predicted by the PolyPhen2, MutPred2, SIFT (sorting intolerant from tolerant) and Mutation Taster methods.

Mutation	PolyPhen2				MutPred 2		SIFT		Mutation Taster
	HumDiv		HumVar						
	Prediction	Score	Prediction	Score	Prediction	Score	Prediction	Score	Prediction
*VWF* c.4381G>C (p.Ala1461Pro)	probably damaging	0.999	probably damaging	0.990	borderline	0.504	pathogenic	0.00	disease causing
*VWF* c.8149G>A (p.Asp2717Asn)	benign	0.000	benign	0.000	benign	0.108	benign	0.43	disease causing
*VWF* c.4141 A>G (p.Thr1381Ala) (rs216311)	benign	0.000	benign	0.001	benign	0.066	benign	0.98	polymorphism
*FGG* c.1085T>A (p.Met362Lys)	probably damaging	1.000	probably damaging	0.999	pathogenic	0.960	pathogenic	0.04	disease causing
*MADH4* c.7A>G (p.Asn3Asp)	benign	0.003	benign	0.002	benign	0.269	pathogenic	0.00	disease causing
*RASA1* c.1833T>G (p.Phe611Leu)	benign	0.014	benign	0.017	pathogenic	0.570	benign	0.31	disease causing

The mutations can be regarded to be probably benign or probably pathogenic if the score value is below or above the 0.5 cut-off value in the case of PolyPhen2 and MutPred2 methods. The mutations can be regarded to be probably pathogenic if the score value is below the 0.05 cut-off value in the case of the SIFT method.

**Table 4:** Detected mutations in fibrinogen disorders.

Fibrinogen Disorders Type	No. Cases (No. Families)	Mutation Type	Gene	Exon	Mutation	
dysfibrinogenemia	1 (1)	missense	FGA	2	c.103C>T	p.Arg35Cys
	8 (4)	missense	FGA	2	c.104G>A	p.Arg35His
	2 (1)	missense	FGA	2	c.116G>C	p.Arg38Ser
	2 (1)	missense	FGB	4	c.586C>T	p.Arg196Cys
	4 (3)	missense	FGG	8	c.902G>A	p.Arg301His
	1 (1)	missense	FGA	5	c.991A>G	p.Thr331Ala
hypofibrinogenemia	4 (1)	missense	FGB	7	c.1067A>G	p.Tyr356Cys
other phenotype or previously not described mutations	2 (1)	missense	FGA	5	c.1634A>T	p.Glu545Val
	1 (1)	nonsense	*FGB*	8	**c.1421G>A**	**p.Trp474Ter**
	1 (1)	missense	*FGG*	8	**c.1085T>A**	**p.Met362Lys**

Novel mutations are indicated in Bold.

## Data Availability

The datasets generated for this study are available on request to the corresponding author.

## Supplementary Materials

The following are available online at https://www.mdpi.com/2075-1729/11/3/202/s1, Table S1: Genotype-phenotype correlations in patients with quantitative types of vWD, Table S2: Genotype-phenotype correlations in patients with qualitative types of vWD, Table S3: Genotype-phenotype correlations in patients with fibrinogen disorders.
