# Supplementary material for: Resolving Differential Diagnostic Problems in von Willebrand Disease, in Fibrinogen Disorders, in Prekallikrein Deficiency and in Hereditary Hemorrhagic Telangiectasia by Next-Generation Sequencing
Source: Life (Basel). 2021 Mar 5;11(3):202. doi: 10.3390/life11030202 (PMC7999415; doi:10.3390/life11030202)
Supplement: Supplementary file 1 [file life-11-00202-s001.pdf]

Supplementary

Supplementary materials 1. Detailed description of clinical cases.

Case 1. vWD patient initially diagnosed as immune thrombocytopenic purpura (ITP); patient 7/1 in Supplementary table 2.

The 59-years-old female patient had low platelet counts in her laboratory files since childhood with the same finding in her mother, who died from intracranial hemorrhage at the age of 26 years, 6 weeks after delivery and who was diagnosed as ITP. The patient had severe epistaxis in her early case history and due to low platelet count she was administered steroid without any effect. At the age of 9, she had splenectomy with only a transient effect on platelet count, which was between 20-30 G/L. She stopped steroid and switched to eltrombopag, a thrombopoietin receptor analog but due to LDH elevation it was discontinued. Clinically she was a bleeder with frequent nose bleedings and heavy menstruation requiring hospitalization. At the age of 45, peripheral arterial occlusion (a. iliaca externa, a. iliaca communis, a. femoralis spf. and profunda), was diagnosed. Aorto-bifemoral bypass surgery was not executed due to the low platelet count, which was reported as 9 G/L at that time, and romiplostim, another thrombopoietin receptor analog was tried, but, again had to be stopped due to LDH elevation. Diagnosis of thrombotic thrombocytopenic purpura (TTP) also emerged. Upon referral to our laboratory, 12-17 G/L platelet count was determined from either EDTA-, citrate-, or heparin- anticoagulated blood samples with platelet aggregates in the peripheral smear; individual platelets, however, were not giant. vWF:Ac to vWF:Ag was very low, however vWF:Ag was elevated due to chronic inflammation on her legs. RIPA showed enhanced aggregation at low dose ristocetin, which was repeatable in the presence of control platelets and the patient's plasma suggesting vWD 2B. No HMWM was detected.

Case 2. Dysfibrinogenemia combined with antithrombin (AT) deficiency. Family 2 in Supplemetary Table 3.

The proband was a 4 years old girl without any symptoms but having a prolonged TT upon routine laboratory check-up before tonsillectomy. She was a heterozygous carrier of p.Arg35His, and wild type for the antithrombin (AT) mutation (see below). She inherited dysfibrinogenemia from her father who was double heterozygous for the FGA mutation and for AT Padua (p.Arg79His) and has had no bleeding or thrombosis, so far. Upon family screening for dysfibrinogenemia the p.Arg35His was found in the paternal grandfather (no symptoms), however, the paternal grandmother, who suffered unprovoked pulmonary embolism at the age of 53 years, no mutation in fibrinogen genes was found. Instead, upon thrombophilia screening a known, type II heparin-binding site AT deficiency (AT Padua) was revealed, which explained the thrombotic phenotype. This finding was the reason, why the whole family was later investigated for AT deficiency. In the laboratory, the most alarming sign for fibrinogen disorder was the prolongation of TT, while PT showed only a slight prolongation, if any and APTT was normal or borderline.

Case 3. Hypofibrinogenemia combined with mutations in F7 and F5 genes. Family 11 in Supplementary Table 3.

The family was investigated due to a young male patient with a complex clinical phenotype of bleeding and thrombosis. Coagulation screening tests of the index patient were normal, or showed borderline values. RT was slightly prolonged. Laboratory phenotype corresponded to hypofibrinogenemia and similar phenomenon was seen in the family members. Causative mutation was found in the family (*FGB* p.Tyr356Cys) in heterozygous form, which is associated with bleeding according to available databases. Since the index patient and family members had borderline coagulation factor VII activities, *F7* gene was additionally analyzed and variations responsible for the lower factor VII levels were found. Analysis of *F5* gene (included originally in our NGS panel) re-

vealed two variants known to associate with increased thrombosis risk (p.Lys858Arg and p.Met2148Thr) and another mutation with factor V lowering effect (p.Met1764Val) in the proband, all of them in heterozygous form. These results may explain the laboratory finding of slightly decreased factor V activity (66%, reference interval 70-120%). Factor V of family members were within the reference interval and genetic analysis showed no mutation at p.Met1764Val.

Case 4. Hypofibrinogenemia with the novel FGB p.Trp474Ter mutation. Patient 13/1 in Supplementary Table 3.

Genetic testing revealed a novel mutation in *FGB* (p.Trp474Ter) in heterozygous form in a female patient with bleeding symptoms. She had prolonged bleedings after dental intervention and heavy menstruation in her case history. She had two amnionic sac hemorrhages at week 7th and 8th of her pregnancies leading to miscarriages, however she had three successful pregnancies afterwards. She delivered her babies with cesarean sections. At the third occasion she was treated with desmopressin to prevent bleeding and a superficial thrombophlebitis developed in her right femoral region. Recently, she was operated due to cervical hernia and fibrinogen (Haemocomplettan P, CSL Behring, Marburg, Germany) was administered because of low fibrinogen levels during routine laboratory check-up. After fibrinogen administration, a left arm superficial thrombophlebitis was registered. Multiple thrombophlebitis events were also described in her mother. In our laboratory, coagulation screening tests showed normal values and fibrinogen was only moderately and proportionally decreased by both the Clauss and the immunological method. FVIII activity was only slightly above the lower limit of reference interval (69%), however, parameters reflecting to vWD were all normal. Platelet aggregation and secretion studies showed no alterations. Inherited thrombophilia was ruled out, since antithrombin, protein C and S levels were all normal, she was not a carrier of FV Leiden mutation and prothrombin 20210A allele. NGS testing found no alterations within the investigated genes, except for the novel, nonsense *FGB* mutation.

Table S1. Genotype-phenotype correlations in patients with quantitative types of vWD.

	**ID**	**Gender (age in years)**	**VWF:Ag 50 -160 %**	**VWF:Ac 61-179 %**	**VWF:CB 60-130 %**	**FVIII 60-150 % (VWF:FVIIIB >40%)**	**RIPA**	**SDS Electro-phoresis for HMWM**	**Symptoms**	**vWF Domain**	**Sequencing results**
					**Mutations associated with vWD type 1**						
1	**1/1**	F (37)	38	40	30	69	ND	ND	easy bruising	D1 A3	E6: *c.657+2T>C HeZ* and E30: c.5278G>A (p.Val1760Ile) HeZ[1]
2	**2/1**	F (45)	32	29	ND	63	N	Normal distribu-tion	no	D2	E11: *c.1187delT (p.Phe396Serfs) HeZ and c.1173_1183delAGGTCAATCAC (p.Thr391delinsLeufs) HeZ*
3	**3/1**	F (29)	42	33	ND	47	↓	Normal distribu-tion	menorrhagia	A2	E28: c.4751A>G (p.Tyr1584Cys) HeZ[2]
4	**4/1**	M (42)	36	46	ND	67	ND	ND	no	D4	E34: c.5768T>C (p.Leu1923Pro) HeZ[3]
5	**5/1**	F (31)	40	34	36	63	ND	Normal distribu-tion	retinal bleeding	A1	E28: c.4141A>G (p.Thr1381Ala) HoZ[4] *(polymorphism)*
					**Mutations associated with vWD type 3**						
6	**6/1**	F (32) (index)	<3	<10	ND	1	ND	ND	menorrhagia, easy bruising	D’	E18: c.2435delC (p.Pro812ArgfsTer31) HoZ[5]
7	**6/2**	F (63) (mother)	62	42	ND	83	ND	ND	no	D’	E18: c.2435delC (p.Pro812ArgfsTer31) HeZ[5]
8	**7/1**	F (55)	20*	<4	ND	<1	ND	ND	menorrhagia, bleeding fol-lowing trau-ma/surgery	D3	E25: c.3379+1G>A HoZ[6]
9	**8/1**	F (32)	<3	<4	ND	4	↓↓	ND	menorrhagia, gum bleeding, muscle hema-toma	D3	E25: c.3379+1G>A HoZ[6]

*Values for vWF:Ag are influenced by Rheumatoid Factor positivity. HeZ, heterozygote; HoZ, homozygote; ND, no data; N, normal RIPA tests results; ↓, decreased PRP aggregation induced by 1.2 mg/mL ristocetin. Novel mutations are indicated in Italics.

Table S2. Genotype-phenotype correlations in patients with qualitative types of vWD.

	ID	Gender (age in years)	VWF:Ag 50 -160 %	VWF:Ac 61-179 %	VWF:CB 60-130 %	FVIII 60-150 % (VWF:FVIIIB >40%)	RIPA	SDS Electro-phoresis for HMWM	Symptoms	vWF Domain	Sequencing results
									**Mutations associated with vWD type 2A**		
**1**	**1/1**	**F (24)**	**58**	**40**	**ND**	92	N	No HMWM detected	no	D3	E22: c.2926C>T (p.Arg976Cys) HeZ[7]
2	**2/1**	M (44) (index)	77	70	65	32 (80)	ND	No HMWM detected	epistaxis, bleeding fol-lowing tooth extraction, easy bruising	D3	E22: c.2926C>T (p.Arg976Cys) HeZ[7]
3	**2/2**	M (48) (brother)	85	71	79	34 (123)	ND	No HMWM detected	epistaxis, bleeding fol-lowing tooth extraction, easy bruising	D3	E22: c.2926C>T (p.Arg976Cys) HeZ[7]
4	**3/1**	M (32) (index)	27	15	21	34	↓	No HMWM detected	bleeding fol-lowing surgery	A2	E28: c.4508T>C (p.Leu1503Pro) HeZ[8]
5	**3/2**	M (36) (brother)	28	14	20	34	↓	No HMWM detected	ND	A2	E28: c.4508T>C (p.Leu1503Pro) HeZ[8]
6	**3/3**	F (61) (mother)	37	19	22	43	↓	No HMWM detected	undefined bleeding	A2	E28: c.4508T>C (p.Leu1503Pro) HeZ[8]
7	**4/1**	F (24) (index)	35	13	ND	50	↓	No HMWM detected	haemoptoe, gum bleeding, bleeding fol-lowing surgery, menorrhagia	A2	E28: c.4628C>T (p.Ser1543Phe) HeZ[9]
8	**4/2**	F (25) (sister)	50	15	ND	109	↓	No HMWM detected	menorrhagia	A2	E28: c.4628C>T (p.Ser1543Phe) HeZ[9]
9	**4/3**	M (21) (brother)	30	11	ND	37	↓	No HMWM detected	epistaxis, bleeding fol-lowing trauma	A2	E28: c.4628C>T (p.Ser1543Phe) HeZ[9]

10	**4/4**	M (56) (father)	48	19	ND	44	↓	No HMWM detected	ND	A2	E28: c.4628C>T (p.Ser1543Phe) HeZ[9]
11	**4/5**	F (48) (mother)	145	122	ND	91	N	Normal distribution	no	A2	E28: c.4628C>T (p.Ser1543Phe) WT
12	**4/6**	F (16) (sister)	52	16	ND	48	ND	No HMWM detected	epistaxis, cutaneous bleeding		no DNA sample available
13	**4/7**	M (14) (brother)	24	12	ND	34	ND	No HMWM detected	epistaxis		no DNA sample available
14	**4/8**	M (19) (brother)	99	120	ND	98	ND	ND	no	A2	E28: c.4628C>T (p.Ser1543Phe) WT
15	**5/1**	F (18)	12	7	6	37	↓ ↓	No HMWM detected	epistaxis, gum bleeding, easy bruising, menorrhagia	A2	E28: c.4789C>T (p.Arg1597Trp) HeZ[10]
16	**6/1**	F (13)	40	13	ND	148	↓	No HMWM detected	epistaxis	A2	E28: c.4883T>C (p.Ile1628Thr) HeZ[11]
**Mutations associated with vWD type 2B**											
17	**7/1**	F (59)	239	31	56	135	↑	No HMWM detected	epistaxis, menorrhagia	D2 A1	E15: c.1781C>G (p.Ala594Gly) HeZ[12] and E28: *c.4381G>C (p.Ala1461Pro) HeZ*
18	**8/1**	F (34)	42	47	51	76	↑	Normal distribution	bleeding following surgery, gum bleeding	D3	E28: c.3797C>T (p.Pro1266Leu) HeZ[13]
19	**9/1**	F (51)	58	49	ND	43	↑	Normal distribution	epistaxis, menorrhagia, postpartum hemorrhage	D3	E28: c.3797C>T (p.Pro1266Leu) HeZ[13]
20	**10/1**	F (21) (index)	125	104	ND	147	↑	Normal distribution	menorrhagia	D3	E28: c.3797C>T (p.Pro1266Leu) HeZ[13]
21	**10/2**	M (49) (father)	127	101	ND	102	↑	Normal distribution	no	D3	E28: c.3797C>T (p.Pro1266Leu) HeZ[13]
22	**10/3**	F (42) (mother)	140	136	ND	110	N	Normal distribution	no	D3	E28: c.3797C>T (p.Pro1266Leu) WT

23	**11/1**	M (46)	80	73	91	96	↑	Normal distribution	bleeding following tooth extraction	D3	E28: c.3797C>T (p.Pro1266Leu) HeZ[13]
24	**12/1**	M (75)	25	13	ND	33	↑	No HMWM detected	umbilical bleeding, epistaxis, bleeding following trauma, GI bleeding	A1	E28: c.3917G>C (p.Arg1306Pro) HeZ[14]
25	**13/1**	F (44)	47	17	16	57	ND	No HMWM detected	epistaxis, gum bleeding, menorrhagia, hematuria, easy bruising	A1	E28: c.3946G>A (p.Val1316Met) HeZ[15]
**Mutations associated with vWD type 2M**											
26	**14/1**	M (70) (index)	88/271*	14	14	18	ND	ND	hematuria	D3	E27: c.3614G>A (p.Arg1205His) HeZ[16]
27	**14/2**	F (44) (daughter)	12	19	ND	27	ND	ND	epistaxis, gum bleeding, menorrhagia, post-partum and post-operative bleeding	D3	E27: c.3614G>A (p.Arg1205His) HeZ[16]
28	**15/1**	F (60)	15	20	ND	23	ND	ND	menorrhagia, bleeding following trauma and tooth extraction	D3	E27: c.3614G>A (p.Arg1205His) HeZ[16]
29	**16/1**	F (76)	8	<10	ND	12	ND	Normal distribution	menorrhagia, post-partum hemorrhage, bleeding following tooth extraction,	D3	E27: c.3614G>A (p.Arg1205His) HeZ[16]

									muscle hematoma		
30	**17/1**	F (67)	7	16	12	7	ND	Normal distribution	bleeding following surgery, menorrhagia	D3	E27: c.3614G>A (p.Arg1205His) HeZ[16]
31	**18/1**	F (34)	13	10	7	18	↓	Normal distribution	epistaxis, menorrhagia	D3	E27: c.3614G>A (p.Arg1205His) HeZ[16]
32	**19/1**	M (4)	17	5	12	36	↓	Normal distribution	bleeding following surgery, gum bleeding	A1 CK	E28: c.3887T>C (p.Leu1296Pro) HeZ[17] and E50: *c.8149G>A (p.Asp2717Asn) HeZ*
**Mutations associated with vWD type 2N or Haemophilia A**											
33	**20/1**	F (33)	62	52	61	14 (37)	ND	ND	epistaxis	D′ D3	E18: c.2365A>G (p.Thr789Ala) HeZ[18] and E21: c.2771 G>A (p.Arg924Gln) HeZ[19]
34	**21/1**	M (16) (index)	39	41	44	18 (13)	ND	Normal distribution	bleeding following surgery	D2 D′ D′	E17: c.2269_70delCT, p.Leu757Valfs*22 HeZ[20]; E18: c.2365A>G (p.Thr789Ala) HeZ[18] and E20: c.2561G>A (p.Arg854Gln) HeZ[21]
35	**21/2**	F (9) (sister)	119	145	151	107	ND	ND	no	D′ D′	E18: c.2365A>G (p.Thr789Ala) HeZ[18] and E20: c.2561G>A (p.Arg854Gln) HeZ19
36	**21/3**	M (13) (brother)	88	113	121	96	ND	ND	no	D′ D′	E18: c.2365A>G (p.Thr789Ala) HeZ[18] and E20: c.2561G>A (p.Arg854Gln) HeZ[21]
37	**21/4**	F (43) (mother)	46	45	51	78 (110)	ND	Normal distribution	ND	D2	E17: c.2269_70delCT, p.Leu757Valfs*22 HeZ[20]
38	**21/5**	M (44) (father)	113	128	151	74	ND	ND	no	D′ D′	E18: c.2365A>G (p.Thr789Ala) HeZ[18] and

											E20: c.2561G>A (p.Arg854Gln) HeZ[21]
39	**21/6**	F (68) (maternal grandmother)	39	39	42	13 (14)	ND	Normal distribution	undefined bleeding	D2<br>D′<br>D′	E17: c.2269_70delCT, p.Leu757Valfs*22 HeZ[20]; E18: c.2365A>G (p.Thr789Ala) HeZ[18] and E20: c.2561G>A (p.Arg854Gln) HeZ[21]
40	**22/1**	F (18) (index)	16	17	24	8 (14)	N	Normal distribution	no	D′<br>D3	E20: c.2561G>A (p.Arg854Gln) HeZ[21] and E25: c.3379+1 G>A HeZ[6]
41	**22/2**	M (15) (brother)	147	100	117	119 (67)	ND	Normal distribution	no	D′<br>D′	E18: c.2365A>G (p.Thr789Ala) HeZ[18] and E20: c.2561G>A (p.Arg854Gln) HeZ[21]
42	**23/1**	F (15) (index)	96	107	151	19 (4)	N	Normal distribution	no	D′<br>D′<br>D′	E18: c.2365A>G (p.Thr789Ala) HeZ[18]; E18: c.2384 A>G (p.Tyr795Cys) HeZ[22] and E20: c.2561G>A (p.Arg854Gln) HeZ[21]
43	**23/2**	M (55) (father)	219	218	221	132 (43)	N	Normal distribution	no	D′	E18: c.2384A>G (p.Tyr795Cys) HeZ[22]
44	**23/3**	F (50) (mother)	70	69	104	51 (52)	N	Normal distribution	no	D′<br>D′	E18: c.2365A>G (p.Thr789Ala) HoZ[18] and E20: c.2561G>A (p.Arg854Gln) HeZ[21]
45	**23/4**	F (18) (sister)	149	144	169	115 (57)	N	Normal distribution	no	D′<br>D′	E18: c.2365A>G (p.Thr789Ala) HeZ[18] and E18: c.2384A>G (p.Tyr795Cys) HeZ[22]
46	**24/1**	F (18) (index)	53	53	ND	22 (<1)	N	Normal distribution	no	D′	E20: c.2561G>A (p.Arg854Gln) HoZ[21]
47	**24/2**	F (43)	182	151	ND	93 (15)	ND	Normal distri-	no	D′	E20: c.2561G>A

		(mother)						bution			(p.Arg854Gln) HeZ[21]
48	**24/3**	M (44) (father)	93	94	ND	57 (24)	ND	Normal distribution	no	D′	E20: c.2561G>A (p.Arg854Gln) HeZ[21]
49	**25/1**	M (22)	48	56	64	15 (14)	ND	Normal distribution	epistaxis, bleeding following trauma and surgery	D′	E20: c.2561G>A (p.Arg854Gln) HoZ[21]
50	**26/1**	M (77)	42	41	ND	24 (12)	ND	Normal distribution	epistaxis, CNS bleeding	D′ A3	E20: c.2561G>A (p.Arg854Gln) HeZ[21] and E31: c.5335C>T (p.Arg1779*) HeZ[23]
51	**27/1**	F (18)	67	62	71	65	ND	ND	*chest venous malformation,* bleeding following trauma, easy bruising,		*F8* E8: c.1064G>A (p.Arg355Gln) HeZ[24]
52	**28/1**	M (10)	75	70	63	4	ND	ND	no		*F8* E14: c.2167G>A (p.Ala723Thr) HemiZ[25]
53	**29/1**	F (48)	129	131	123	55 (95)	ND	ND	epistaxis, menorrhagia, bleeding following surgery		*F8* E14: c.4379delA (p.Asn1460fs*5) HeZ[25]
54	**30/1**	M (25)	181	167	ND	3 (127)	ND	Normal distribution	epistaxis, bleeding following tooth extraction, hemarthrosis, muscle hematoma		*F8* E14: c.5122C>T (p.Arg1708Cys) HemiZ[26]

*Values for vWF:Ag are influenced by Rheumatoid Factor positivity. HeZ, heterozygote; HoZ, homozygote; WT, wild type; ND, no data; N, normal RIPA tests results; ↓ , decreased PRP aggregation induced by 1,2 mg/mL ristocetin; ↑ , increased PRP aggregatin induced by 0,6 mg/mL ristocetin Novel mutations are indicated in Italics.

Table S3. Genotype-phenotype correlations in patients with fibrinogen disorders.

	ID	Gender (age in years)	PT 8.4-12.5 sec	APTT 24.2-36.6 sec	TT 15.5-23.7 sec	RT 18.6-26.2 sec	Fng 1.5-4.0 g/L	Fng Ag 1.80-3.50 g/L	Bleeding event	Thrombotic event	Genetic result
**Mutations associated with dysfibrinogenaemia phenotype**											
**1**	1/1	F (72)	14.6	29.3	80.4	>100	<0.50	3.56	prolonged bleeding after dental surgery	-	*FGA*: c.103C>T (p.Arg35Cys) HeZ[27]
**2**	2/1	F (14) (index)	13.9	33.1	63.5	>100	0.66	2.28	-	-	*FGA*: c.104G>A (p.Arg35His) HeZ [28]<br>***SERPINC1*: c.236G>A (p.Arg79His) WT**
**3**	2/2	M (41) (father)	10.4	30.7	42.0	>100	1.05	4.70	-	-	*FGA*: c.104G>A (p.Arg35His) HeZ[28];<br>***SERPINC1*: c.236G>A (p.Arg79His) HeZ**
**4**	2/3	F (44) (mother)	8.9	27.7	17.1	19.5	3.13	2.79	-	-	*FGA*: c.104G>A (p.Arg35His) WT
**5**	2/4	F (63) (paternal grandmoth-er)	31.9*	50.0	16.8	19.2	4.09	4.10	-	PE	*FGA*: c.104G>A (p.Arg35His) WT;<br>***SERPINC1*: c.236G>A (p.Arg79His) HeZ**
**6**	2/5	M (63) (paternal grandfather)	11.4	29.3	51.3	>100	0.80	2.96	-	-	*FGA*: c.104G>A (p.Arg35His) HeZ[28]
**7**	3/1	F (64) (index)	13.1	38.9	62.3	>100	0.50	4.94	-	-	*FGA*: c.104G>A (p.Arg35His) HeZ[28]
**8**	3/2	F (32) (daughter)	11.6	29.8	49.4	>100	0.76	2.42	prolonged bleeding after tooth extrac-tion	-	*FGA*: c.104G>A (p.Arg35His) HeZ[2]
**9**	3/3	F (28) (daughter)	11.8	31.0	44.4	>100	0.78	2.77	prolonged bleeding after tooth extrac-tion	-	*FGA*: c.104G>A (p.Arg35His) HeZ [28]

**10**	4/1	M (16)	14.2	33.2	48.4	>100	0.75	2.83	-	-	*FGA*: c.104G>A (p.Arg35His) HeZ [28]
**11**	5/1	F (56)	10.6	32.8	57.8	75.9	1.30	2.41	mild bruising	DVT	*FGA*: c.104G>A (p.Arg35His) HeZ [28]; *FGA*: c.991A>G (p.Thr331Ala) HeZ [29]; **Factor V Leiden HeZ**
**12**	6/1	F (51) (index)	10.1	27.1	34.0	65.1	0.67	3.36	-	-	*FGA*: c.116G>C (p.Arg38Ser) HeZ [30]
**13**	6/2	F (21) (daughter)	10.6	30.8	36.8	68.9	0.65	2.88	-	-	*FGA*: c.116G>C (p.Arg38Ser) HeZ[30]
**14**	7/1	F (40) (index)	9.1	27.0	17.3	20.4	<0.50	2.85	prolonged bleeding after childbirth	-	*FGB*: c.586C>T (p.Arg196Cys) HeZ [31]; ***VWF* E3: c.101G>A (p.Arg34Gln) HeZ**[32]
**15**	7/2	M (10) (son)	9.5	41.0	18.2	20.2	<0.50	2.18	-	-	*FGB*: c.586C>T (p.Arg196Cys) HeZ [31]; ***VWF* E3: c.101G>A (p.Arg34Gln) WT**
**16**	8/1	F (49) (index)	10.0	25.3	35.6	41.2	0.55	2.80	epistaxis, bleeding after cesarean section, placental hemorrhage	-	*FGG*: c.902G>A (p.Arg301His) HeZ [33]
**17**	8/2	M (19) (son)	10.2	29.0	37.4	40.8	1.31	2.29	-	-	*FGG*: c.902G>A (p.Arg301His) HeZ [33]
**18**	9/1	F (47)	11.6	29.2	44.6	60.9	1.41	1.95	epistaxis	-	*FGG*: c.902G>A (p.Arg301His) HeZ [33]
**19**	10/1	M (63)	10.0	33.4	36.4	41.5	1.45	3.49	-	-	*FGG*: c.902G>A (p.Arg301His) HeZ [33]
**Mutations associated with hypofibrinogenaemia phenotype**											

**20**	11/1	M (20)<br>(index)	12.0	27.4	24.0	31.4	0.98	0.95	epistaxis, bleeding gums	sagittal sinus thrombosis	*FGB*: c.1067A>G (p.Tyr356Cys) HeZ [34];<br>***F7*: c.-323 10 nucleotide insertion, c. -122T>C and c.1241G>A (p.Arg413Gln) HeZ;**<br>***F5*: c.2573A>G (p.Lys858Arg) and c.5290A>G (p.Met1764Val) and c.6443T>C (p.Met2148Thr) HeZ**
**21**	11/2	F (14)<br>(sister)	10.8	27.4	21.1	ND	1.30	1.26	**-**	**-**	*FGB*: c.1067A>G (p.Tyr356Cys) HeZ[34];<br>***F7*: c.-323 10 nucleotide insertion. c. -122T>C and c.1241 G>A (p.Arg413Gln) HeZ;**<br>***F5*: c.2573A>G (p.Lys858Arg) and c.5290A>G (p.Met1764Val) HeZ and c.6443T>C (p.Met2148Thr) WT**
**22**	11/3	M (26)<br>(brother)	9.8	27.5	19.4	ND	1.80	1.62	epistaxis, prolonged bleeding after tooth extraction	**-**	*FGB*: c.1067A>G (p.Tyr356Cys) HeZ [34];<br>***F7*: c.-323 10 nucleotide insertion. c. -122T>C and c.1241 G>A (p.Arg413Gln) HeZ;**<br>***F5*: c.2573A>G (p.Lys858Arg) and c.5290A>G (p.Met1764Val) HeZ and c.6443T>C (p.Met2148Thr) WT**
**23**	11/4	F (48)<br>(mother)	9.2	25.2	19.9	ND	1.88	1.86	**-**	**-**	*FGB*: c.1067A>G (p.Tyr356Cys) HeZ [34];<br>***F7*: c.-323 10 nucleotide insertion. c. -122T>C and c.1241 G>A (p.Arg413Gln) HeZ;**<br>***F5*: c.2573A>G (p.Lys858Arg) and c.5290A>G (p.Met1764Val) HeZ and c.6443T>C (p.Met2148Thr) WT**
**Other phenotype or previously not described mutation**											
**24**	12/1	M (65)<br>(index)	7.9	27.2	14.2	ND	2.60	ND	-	-	*FGA*: c.1634A>T (p.Glu545Val) HeZ [35]

**25**	12/2	M (67) (cousin)	7.4	27.7	18.5	ND	ND	ND	-	-	*FGA*: c.1634A>T (p.Glu545Val) HeZ [35]
**26**	13/1	F (42)	11.7	34.6	22.9	ND	0.83	0.82	prolonged bleeding after tooth extraction, heavy menstruation, 2 miscarriages	Post-treatment hrombophlebitis	*FGB: c.1421G>A (p.Trp474*) HeZ*
**27**	14/1	M (61)	10.9	27.2	29.8	33.2	<0.50	1.38	-	stroke	*FGG: c.1085T>A (p.Met362Lys) HeZ*

HeZ, heterozygote; HoZ homozygote; WT, wild type for family mutation; DVT, deep vein thrombosis; PE, pulmonary embolism. Coagulation factor VII activity values for patients 20, 21, 22 and 23 were 50%, 72%, 68% and 75%, respectively. Reference interval for factor VII activity 70-120%. *The patient was on vitamin K antagonist therapy at the time of blood sampling.

References for Table S1:

1. James, P.D.; Notley C., Hegadorn, C.; Leggo, J.; Tuttle, A.; Tinlin, S.; Brown, C.; Andrews, C.; A Labelle, A.et al. The mutational spectrum of type 1 von Willebrand disease: Results from a Canadian cohort study, *Blood*. **2007**, *109*, 145–154
2. O'Brien, L.A.; James, P. D.; Othman, M.; Berber, E.; Cameron, C.; Notley, C.R.P.; Hegadorn, C.A.; Sutherland, J.J.; Hough, C.; Rivard, G.E. et al. Founder von Willebrand factor haplotype associated with type 1 von Willebrand disease, *Blood*. **2003**, *102*, 549–557.
3. Johansson, A.M.; Halldén, C.; Säll, T.; Lethagen, S. Variation in the VWF gene in Swedish patients with type 1 von Willebrand Disease, *Ann Hum Genet.* **2011**, *75*, 447–455.
4. Szántó, T.; Schlammadinger, A.; Staelens, S.; Meyer, S.F.D.; Freson, K.; Pareyn, I.; Vauterin, S.; Hársfalvi, J.; Deckmyn, H.; anhoorelbeke,K.V. The A/T1381 polymorphism in the A1-domain of von Willebrand factor influences the affinity of von Willebrand factor for platelet glycoprotein Ibalpha, *Thromb Haemost.* **2007**, *98*, 178–185.
5. Zhang, Z.P.; Falk, G.; Blombäck, M.; Egberg, N.; Anvret, M. A single cytosine deletion in exon 18 of the von Willebrand factor gene is the most common mutation in Swedish vWD type III patients, *Hum Mol Genet.* **1992**, *1*, 767–768.
6. Nesbitt, I.M.; Hampton, K.K.; Preston,F.E.; Peake, I.R.; Goodeve, A.C. A common splice site mutation is shared by two families with different type 2N von Willebrand disease mutations, *Thromb Haemost.* **1999**, *82*, 1061–1064.

References for Table S2.

7. Schneppenheim, R.; Michiels, J.J.; Obser, T.; Oyen, F.; Pieconka, A.; Schneppenheim, S.; Will, K.; Zieger, B.; Budde, U. A cluster of mutations in the D3 domain of von Willebrand factor correlates with a distinct subgroup of von Willebrand disease: type 2A/IIE, *Blood*. **2010**, *115*, 4894–4901.
8. Jacques Michiels, J.; van Vliet, H.H.D.M. Dominant von Willebrand disease type 2A groups I and II due to missense mutations in the A2 domain of the von Willebrand factor gene: diagnosis and management, *Acta Haematol.* **2009**, *121*, 154–166.
9. Meyer, D.; Fressinaud, E.; Gaucher, C.; Lavergne, J.M.; Hilbert, L.; Ribba, A.S.; Jorieux, S.; Mazurier, C. Gene defects in 150 unrelated French cases with type 2 von Willebrand disease: from the patient to the gene. INSERM Network on Molecular Abnormalities in von Willebrand Disease, *Thromb Haemost.* **1997**, *78*, 451–456.
10. Ginsburg, D.; Konkle, B.A.; Gill, J.C.; Montgomery, R.R.; Bockenstedt, P.L.; Johnson, T.A.; Yang, A.Y. Molecular basis of human von Willebrand disease: analysis of platelet von Willebrand factor mRNA, *Proc Natl Acad Sci U S A.* **1989**, *86*, 3723–3727.
11. Iannuzzi, M.C.; Hidaka, N.; Boehnke, M.; Bruck, M.E.; Hanna, W.T.; Collins, F.S.; Ginsburg, D. Analysis of the relationship of von Willebrand disease (vWD) and hereditary hemorrhagic telangiectasia and identification of a potential type IIA vWD mutation (IIe865 to Thr), *Am J Hum Genet.* **1991**, *48*, 757–763.
12. Corrales, I.; Ramírez, L.; Altisent, C.; Parra, R.; Vidal, F. Rapid molecular diagnosis of von Willebrand disease by direct sequencing. Detection of 12 novel putative mutations in VWF gene, *Thromb Haemost.* **2009**, *101*, 570–576.
13. Holmberg, L.; Dent, J.A.; Schneppenheim, R.; Budde, U.; Ware, J.; Ruggeri, Z.M. von Willebrand factor mutation enhancing interaction with platelets in patients with normal multimeric structure, *J Clin Invest.* **1993**, *91*, 2169–2177.
14. Veyradier, A.; Boisseau, P.; Fressinaud, E.; Caron, C.; Ternisien, C.; Giraud, M.; Zawadzki, C.; Trossaert, M.; Itzhar-Baïkian, N.; Dreyfus, M. A Laboratory Phenotype/Genotype Correlation of 1167 French Patients From 670 Families With von Willebrand Disease: A New Epidemiologic Picture, *Medicine (Baltimore)*. **2016**, *95*, e3038.
15. Randi, A.M.; Rabinowitz, I.; Mancuso, D.J.; Mannucci, P.M.; Sadler, J.E. Molecular basis of von Willebrand disease type IIB. Candidate mutations cluster in one disulfide loop between proposed platelet glycoprotein Ib binding sequences, *J Clin Invest*. **1991**, *87*, 1220–1226.
16. Schneppenheim, R.; Federici, A.B.; Budde, U.; Castaman, G.; Drewke, E.; Krey, S.; Mannucci, P.M.; Riesen, G.; Rodeghiero, F.; Zieger, B.; Zimmermann, R. Von Willebrand Disease type 2M "Vicenza" in Italian and German patients: identification of the first candidate mutation (G3864A; R1205H) in 8 families, *Thromb Haemost*. **2000**, *83*, 136–140.
17. Meyer, D.; Fressinaud, E.; Gaucher, C.; Lavergne, J.M.; Hilbert, L.; Ribba, A.S.; Jorieux, S.; Mazurier, C. Gene defects in 150 unrelated French cases with type 2 von Willebrand disease: from the patient to the gene. INSERM Network on Molecular Abnormalities in von Willebrand Disease, *Thromb Haemost*. **1997**, *78*, 451–456.
18. Ahmad, F.; Kannan, M.; Biswas, A.; Saxena, R. Impact of 789Ala/Ala genotype on quantitative type of von Willebrand disease, *Ann Hematol.* 2009, *88*, 479–483.

19. Casais, P.; Carballo, G.A.; Woods, A.I.; Kempfer, A.C.; Farías, C.E.; Grosso, S.H.; Lazzari, M.A. R924Q substitution encoded within exon 21 of the von Willebrand factor gene related to mild bleeding phenotype, *Thromb Haemost.* **2006**, *96*, 228–230.
20. Baronciani, L.; Cozzi, G.; Canciani, M.T.; Peyvandi, F.; Srivastava, A.; Federici, A.B.; Mannucci, P.M. Molecular defects in type 3 von Willebrand disease: updated results from 40 multiethnic patients, *Blood Cells Mol Dis.* **2003**, *30*, 264–270.
21. Gaucher, C.; Mercier, B.; Jorieux, S.; Oufkir, D.; Mazurier, C. Identification of two point mutations in the von Willebrand factor gene of three families with the 'Normandy' variant of von Willebrand disease, *Br J Haematol.* **1991**, *78*, 506–514.
22. Schneppenheim, R.; Budde, U.; Obser,T.; Brassard, J.; Mainusch, K.; Ruggeri, Z.M.; Schneppenheim, S.; Schwaab, R.; Oldenburg, J. Expression and characterization of von Willebrand factor dimerization defects in different types of von Willebrand disease, *Blood*. **2001**, *97*, 2059–2066.
23. Goodeve, A.; Eikenboom, J.; Castaman, G.; Rodeghiero, F.; Federici, A.B.; Batlle, J.; Meyer, D.; Mazurier, C.; Goudemand, J.; Schneppenheim, R. Phenotype and genotype of a cohort of families historically diagnosed with type 1 von Willebrand disease in the European study, Molecular and Clinical Markers for the Diagnosis and Management of Type 1 von Willebrand Disease (MCMDM-1VWD), *Blood*. **2007**, *109*, 112–121.
24. Lu, Y.; Xin, Y.; Dai, J.; Wu, X.; You, G.; Ding, Q.; Wu, W.; Wang, X. Spectrum and origin of mutations in sporadic cases of haemophilia A in China, *Haemophilia*. **2018**, *24*, 291–298.
25. Higuchi, M.; Kazazian Jr, H.H.; Kasch, L.; Warren, T.C.; McGinniss, M.J.; Phillips 3rd, J.A.; Kasper, C.; Janco, R.; Antonarakis, S.E. Molecular characterization of severe hemophilia A suggests that about half the mutations are not within the coding regions and splice junctions of the factor VIII gene, *Proc Natl Acad Sci U S A*. **1991**, *88*, 7405–7409.
26. Gitschier, J.; Kogan, S.; Levinson, B.; Tuddenham, E.G. Mutations of factor VIII cleavage sites in hemophilia A, *Blood*. **1988**, *72*, 1022–1028.

References for Table S3.

27. Galanakis, D.; Spitzer, S.; Scharrer, I. Unusual A alpha 16Arg-->Cys dysfibrinogenaemic family: absence of normal A alpha-chains in fibrinogen from two of four heterozygous siblings, *Blood Coagul Fibrinolysis.* **1993**, *4*, 67–71.
28. Higgins, D.L.; Shafer, J.A. Fibrinogen Petoskey, a dysfibrinogenemia characterized by replacement of Arg-A alpha 16 by a histidyl residue. Evidence for thrombin-catalyzed hydrolysis at a histidyl residue, *J Biol Chem.* **1981**, *256*, 12013–12017.
29. Carter, A.M.; Catto, A.J.; Grant, P.J. Association of the alpha-fibrinogen Thr312Ala polymorphism with poststroke mortality in subjects with atrial fibrillation, *Circulation*. **1999**, *99*, 2423–2426.
30. Tiscia, G.L. and Margaglione, M. Human Fibrinogen: Molecular and Genetic Aspects of Congenital Disorders, *Int J Mol Sci.* **2018**, *19*, 1597.
31. Lounes, K.C.; Lefkowitz, J.B.; Henschen-Edman, A.H.; Coates, A.I.; Hantgan, R.R.; Lord, S.T. The impaired polymerization of fibrinogen Longmont (Bbeta166Arg-->Cys) is not improved by removal of disulfide-linked dimers from a mixture of dimers and cysteine-linked monomers, *Blood*. **2001**, *98*, 661–666.
32. Hampshire, D.J.; Abuzenadah, A.M.; Cartwright, A.; Al-Shammari, N.S.; Coyle, R.E.; Eckert, M.; Al-Buhairan, A.M.; Messenger, S.L.; Budde, U.; Gürsel, T. Identification and characterisation of mutations associated with von Willebrand disease in a Turkish patient cohort, *Thromb Haemost*. **2013**, *110*, 264–274.
33. Borrell, M.; Garí, M.; Coll, I.; Vallvé, C.; Tirado, I.; Soria, J.M.; Sala, N.; Muñoz, C.; Oliver, A.; García, A. et al. Abnormal polymerization and normal binding of plasminogen and t-PA in three new dysfibrinogenaemias: Barcelona III and IV (gamma Arg 275-->His) and Villajoyosa (gamma Arg 275-->Cys), *Blood Coagul Fibrinolysis*. **1995**, *6*, 198–206.
34. Hanss, M.; Chevreaud, C.; French, P.; Négrier, C.; de Mazancourt, P. Clinical and biological features of 3 cases of hypofibrinogenemia associated with three different mutations (gamma Ala341Thr, Bbeta Tyr326Cys and Aalpha Asp496Asn), *Thromb Haemost*. **2007**, *98*, 689–691.
35. Uemichi, T.; Liepnieks, J.J.; Benson, M.D. Hereditary renal amyloidosis with a novel variant fibrinogen, *J Clin Invest*. 1994, *93*, 731–736.
